# Molecular and Cellular Mechanisms of Myocardial Ischemia and Reperfusion Injury: A Narrative Review

**DOI:** 10.3390/cells15030265

**Published:** 2026-01-30

**Authors:** Stefan Juricic, Jovana Klac, Sinisa Stojkovic, Branko Beleslin, Milorad Tesic, Ivana Jovanovic, Marko Banovic, Olga Petrovic, Srdjan Aleksandric, Natalija Vasic, Filip Simeunovic, Dejan Lazovic, Milica Stoiljkovic, Sashko Nikolov, Dejan Simeunovic

**Affiliations:** 1 Clinic for Cardiology, University Clinical Center of Serbia, 11000 Belgrade, Serbia; stefan.juricic@gmail.com (S.J.); sstojkovi@mts.rs (S.S.); misa.tesic@gmail.com (M.T.); ivana170679@gmail.com (I.J.);; 2 Faculty of Medicine, University of Belgrade, 11000 Belgrade, Serbia; branko.beleslin@gmail.com (B.B.); opetrovic1976@gmail.com (O.P.);; 3 Department of Cardiology, Emergency Center, University Clinical Center of Serbia, 11000 Belgrade, Serbia; 4 Clinic for Cardiac Surgery, University Clinical Center of Serbia, 8th Kosta Todorovic St., 11000 Belgrade, Serbia; lazovic.dejan88@gmail.com; 5 Clinic for Endocrinology, Diabetes and Metabolic Diseases, University Clinical Centre of Serbia, 11000 Belgrade, Serbia; 6 Faculty of Medical Sciences, Goce Delcev University, 2000 Stip, North Macedonia

**Keywords:** myocardial ischemia, reperfusion injury, mitochondrial dysfunction, ROS, apoptosis, necroptosis

## Abstract

Myocardial ischemia represents a state of reduced coronary perfusion with oxygenated blood, insufficient to meet the metabolic demands of the myocardium. Both acute and chronic ischemia trigger a cascade of cellular events that lead to disturbances in ionic balance, mitochondrial function and energy metabolism. During ischemia, cardiomyocytes (CMs) shift from aerobic to anaerobic metabolism, resulting in adenosine triphosphate (ATP) depletion, loss of ionic homeostasis and calcium (Ca^2+^) overload that activate proteases, phospholipases and membrane damage. Reperfusion restores oxygen supply and prevents irreversible necrosis but paradoxically initiates additional injury in marginally viable myocardium. The reoxygenation phase induces excessive production of reactive oxygen species (ROS), endothelial dysfunction and a strong inflammatory response mediated by neutrophils, platelets and cytokines. Mitochondrial dysfunction and opening of the mitochondrial permeability transition pore (mPTP) further amplify oxidative stress and inflammation and trigger apoptosis and necroptosis. Understanding these intertwined cellular and molecular mechanisms remains essential for identifying novel therapeutic targets aimed at reducing reperfusion injury and improving myocardial recovery after ischemic events.

## 1. Introduction

Myocardial ischemia may occur as a consequence of acute or chronic disorders. The main characteristic of these conditions is an imbalance between the myocardial demand for oxygen and nutrients and their availability. Acute myocardial ischemia most commonly occurs due to a sudden reduction or complete interruption of blood flow through the coronary arteries, which directly endangers the function and survival of CMs. If perfusion is not restored promptly, myocardial infarction (MI) may develop. The most common cause of this process is an atherothrombotic event that partially or completely occludes the lumen of a coronary artery [1,2,3].

On the other hand, chronic myocardial ischemia represents a prolonged state of reduced oxygen supply, caused by persistently impaired blood flow through the coronary arteries. Under these conditions, cumulative myocardial damage occurs during resting phases as a result of prolonged compensatory vasodilation. In contrast, during increased metabolic demands, such as physical exertion or stress, ischemia manifests when compensatory dilation of the coronary system fails to meet the CMs oxygen requirements [1,2]. Stable atherosclerotic plaques that significantly reduce coronary perfusion are most commonly responsible for chronic ischemic heart disease.

In acute MI, prompt restoration of blood flow through the coronary arteries is essential for safeguarding the myocardium and mitigating mortality. However, the reperfusion process can also have harmful consequences: it leads to additional myocardial damage known as reperfusion injury, due to excessive production of ROS, Ca^2+^ overload in cells and mitochondrial damage. For this reason, in understanding the pathophysiology of acute ischemia, it is essential to distinguish between the ischemic and reperfusion phases, as the molecular mechanisms of myocardial damage differ significantly between these stages [1,3].

This review outlines the landscape of ischemia-reperfusion (I/R) injury by linking fundamental molecular mechanisms and various types of cell death with their broader clinical and systemic manifestations. In addition to analyzing intracellular processes, the paper integrates the roles of microvascular dysfunction and pericytes, alongside the splenic contribution to the systemic immune response. By aligning these elements, the review aims to trace the trajectory from basic pathophysiological phenomena to current cardioprotective and therapeutic strategies while identifying key questions that remain the subject of intense investigation.

## 2. Materials and Methods

This work represents a narrative literature review aimed at presenting and synthesizing current knowledge on the molecular and metabolic responses during I/R injury of the myocardium, types of cell death, microvascular dysfunction, cardioprotective mechanisms, as well as translational pathways between preclinical and clinical models.

The literature search was conducted in PubMed and Scopus, using keywords including I/R injury, ROS, opening of the mPTP, Ca^2+^ overload, microvascular obstruction (MVO), pericytes and cardioprotection. Studies in English were included, comprising both original studies (preclinical and clinical) and review articles. Animal studies involved rodents, pigs and dogs, while clinical studies included relevant human populations.

The search covered the period from 1974 to 21 April 2025, including older seminal studies that laid the foundations for understanding pathophysiological mechanisms, as well as more recent studies (one-third of publications from the last 5 years) to ensure the review reflects current knowledge and relevance.

Studies were included if they were published in English, original preclinical or clinical studies, or relevant review articles focusing on molecular and metabolic mechanisms of I/R injury, types of cell death, microvascular dysfunction and cardioprotective strategies. Studies were excluded if they were published in other languages, were comments, editorials, or conference abstracts without full text, not directly relevant, had unclear design or incomplete results, or were duplicates. Priority was given to key seminal studies, while more recent studies were used to illustrate current translational findings and research trends.

Since this is a narrative review, the manuscript is not a systematic review and there is a risk of selection bias, which represents one of the limitations of this work.

## 3. Molecular and Metabolic Responses During Myocardial Ischemia-Reperfusion Injury

**Figure 1:**
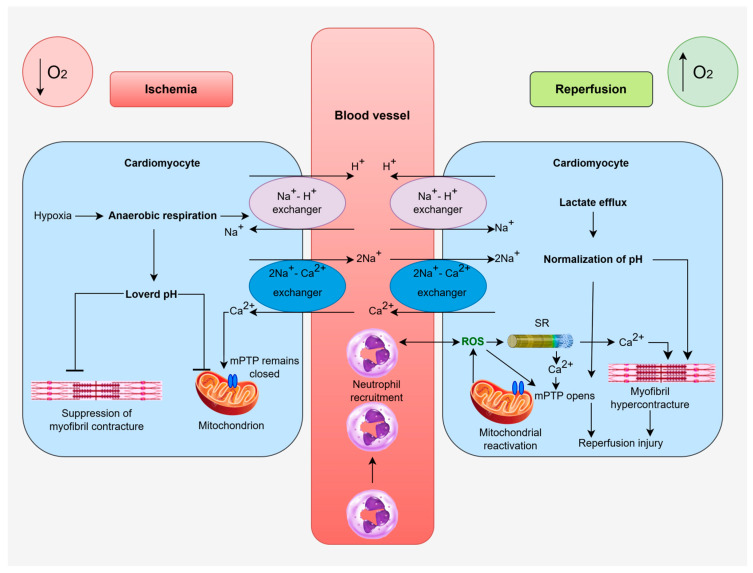
Molecular mechanisms of ischemia/reperfusion injury in cardiomyocytes. The figure illustrates the key molecular mechanisms occurring in CMs during I/R injury. During ischemia, a decline in pH, reduced ATP synthesis, and impaired ion pump function create conditions for mitochondrial Ca^2+^ overload, increased ROS production, and mPTP opening—critical events during the reperfusion phase. Abbreviations: ATP, adenosine triphosphate; CMs, cardiomyocytes; I/R, ischemia/reperfusion; mPTP, mitochondrial permeability transition pore; ROS, reactive oxygen species; SR, sarcoplasmic reticulum.

During myocardial ischemia, due to reduced oxygen supply, the heart muscle shifts to anaerobic metabolism (anaerobic glycolysis). This process is accompanied by rapid consumption of the remaining oxygen and cessation of mitochondrial respiration, resulting in a drastic decline in ATP concentration [4,5,6,7]. As a result of the activation of anaerobic metabolism, lactic acid and hydrogen ions accumulate, causing a decrease in intracellular pH (cellular acidosis) and interfering with the further progression of glycolysis. Cardiomyocytes lose their contractile function, and an early net loss of potassium (K^+^) ions also occurs [4]. Due to the insufficient amount of ATP, anaerobic metabolism is unable to maintain an adequate ionic concentration gradient in the sarcolemma and the mitochondrial membrane potential (ΔΨ_m_). Adenosine triphosphate depletion also causes inactivation of ion pumps, especially the Na^+^/K^+^-ATPase, which leads to K^+^ efflux, Na^+^, Cl^−^ and H_2_O accumulation and consequent cellular swelling. Simultaneously, the Na^+^/Ca^2+^ exchanger (NCX) operates in reverse mode, leading to Ca^2+^ accumulation within the cytosol. These disturbances, along with excessive Ca^2+^ accumulation, lead to a key stage of injury—increased mitochondrial membrane permeability [3,4] (Figure 1).

Consequently, this leads to the release of cytochrome c (Cyt c), activation of caspases and further suppression of ATP synthesis, accompanied by enhanced production of ROS, which progressively damage cellular components [4,8].

This cascade—from ATP depletion and electrolyte imbalance to the degradation of membrane phospholipids—forms the basis of oncotic injury (oncosis), which can progressively lead to necroptosis, pyroptosis, or classical necrosis [4,9,10,11]. Since mature CMs have low expression of proteins necessary for apoptosis, an energy-dependent process, cell death is more likely to occur through necrotic mechanisms, which will be discussed later [4,9].

### 3.1. Calcium Overload and Mitochondrial Dysfunction

Calcium plays a very important role in the pathophysiology of the heart muscle. In the heart outside of ischemia, changes in cytosolic Ca^2+^ concentration induce contraction and relaxation of myofibrils, while mitochondrial Ca^2+^ adjusts the rate of ATP consumption in accordance with the oxidative metabolism of the mitochondria. In I/R injury, one of the main mechanisms responsible for the disruption of myocyte integrity is mitochondrial Ca^2+^ overload. Therefore, current therapeutic strategies are aimed at controlling intracellular Ca^2+^ transport [12].

The main pathway for Ca^2+^ entry into mitochondria is the mitochondrial calcium uniporter (MCU), whose pore-forming component, together with the regulatory subunits EMRE and MICU1-3, controls the ion flow. Mitochondrial calcium uptake 1 functions as a “gatekeeper”: at low Ca^2+^ concentrations, it blocks the pore, while at higher concentrations, it allows rapid Ca^2+^ entry. The proximity of mitochondria and the sarcoplasmic reticulum (SR) allows for local increases in Ca^2+^ to exceed the threshold for MCU entry [12,13].

The dominant pathway for Ca^2+^ extrusion from mitochondria is the mitochondrial NCX, whose activity depends on the Na^+^ gradient across the inner mitochondrial membrane (IMM). The slower extrusion kinetics compared to MCU entry allows a gradual increase in matrix Ca^2+^, stimulation of the tricarboxylic acid (TCA) cycle and preservation of mitochondrial pyridine nucleotides. The balance between MCU entry and NCX extrusion precisely regulates mitochondrial Ca^2+^ and thereby ATP production [12,14].

During myocardial ischemia, reduced oxygen supply halts mitochondrial oxidative metabolism, which decreases the levels of ATP and phosphocreatine needed for normal myocyte contraction and relaxation. As a consequence, the function of ATP-dependent Na^+^ pumps declines, leading to Na^+^ accumulation in the cytosol and K^+^ loss from the cell. Compensatorily, activated anaerobic glycolysis increases the H^+^ concentration in the cytosol, reducing the affinity of the myofilaments for Ca^2+^ and further stimulating Na^+^ entry via the Na^+^/H^+^ exchanger (NHE) [15]. Elevated Na^+^ reverses the operation of the NCX on the sarcolemma, favoring Ca^2+^ entry into the cell. Simultaneously, Ca^2+^ enters through L-type calcium channels, while reduced sarcoplasmic/endoplasmic reticulum Ca^2+^-ATPase activity within the SR limits Ca^2+^ reuptake. This leads to an elevation in cytosolic Ca^2+^ and its subsequent transfer into mitochondria.

Prolonged ATP depletion creates a hyperosmolar environment that causes cell swelling and protein damage, leading to myocyte death [12] (Figure 1).

Mitochondrial Ca^2+^ overload in early reperfusion triggers the opening of the mPTP, leading to the loss of proteins and solutes from the mitochondria, membrane collapse and oxidative stress. The opening of the mPTP and the consequent changes in mitochondria are key causes of I/R injury, which will be discussed in the next section [12].

### 3.2. Opening of the Mitochondrial Permeability Transition Pore

The mPTP is a non-selective channel in the IMM that allows the passage of solutes smaller than 1.5 kDa between the mitochondrial matrix and the cytoplasm. The opening of the mPTP can manifest in two functionally distinct modalities: transient, which is involved in the transport of protons, Ca^2+^ ions, mitochondrial reactive oxygen species (mtROS) and other signaling molecules from the mitochondria to the cytosol; permanent (irreversible), which inhibits ATP production, leads to collapse of ΔΨ_m_, irreversible interruption of oxidative phosphorylation and a necrotic form of cell death. Short-term, reversible opening of the mPTP, which occurs at low conductance, allows the transfer of H^+^ and Ca^2+^ ions across the IMM and the release of signaling molecules, such as Ca^2+^ and mtROS, into the cytosol. This process is known as “flickering mPTP” and causes a temporary depolarization of the ΔΨ_m_, which activates the respiratory chain. It is important to emphasize that this mechanism does not reduce the production of mtROS; instead, it specifically mitigates mtROS accumulation within the mitochondrial matrix. By facilitating the transient efflux of ROS into the cytosol, mPTP flickering acts as a ‘release valve’ that prevents the matrix concentration from reaching the critical threshold required for permanent pore opening and subsequent cell death [16]. In contrast, long-term, irreversible opening of the mPTP causes permanent mitochondrial depolarization, disruption of ion and solute distribution, damage to respiratory chain supercomplexes and increased mtROS production, which can ultimately lead to cell death [17].

Contemporary research indicates that the mPTP does not open during ischemia, but primarily in early reperfusion (Figure 1). During ischemia, mitochondria are in a reduced state with low ΔΨ_m_ and limited ROS production, which prevents the formation of conditions for mPTP activation. Restoration of oxygen flow during early reperfusion increases mtROS production and the recovery of ΔΨ_m_, resulting in rapid Ca^2+^ entry into the mitochondria and the creation of conditions for the mPTP opening [18].

**Figure 2:**
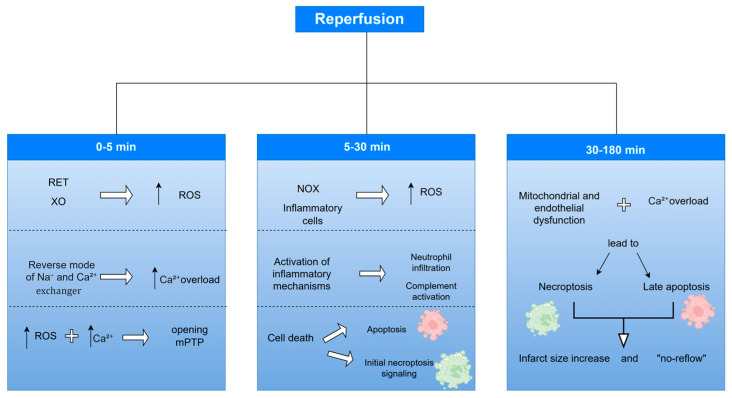
Chronological sequence of molecular events during early reperfusion. The figure represents a time diagram illustrating ionic currents, sources of ROS and the dominance of specific cell death pathways during the early phase of reperfusion. Time frames are based on established data from [19,20,21,22,23,24]. Abbreviations: mPTP, mitochondrial permeability transition pore; NOX, NADPH oxidases; RET, reverse electron transport; ROS, reactive oxygen species; XO, xanthine oxidase.

Excess Ca^2+^ in mitochondria has been recognized as a key cause of irreversible mitochondrial damage and CMs death during I/R injury. Uncontrolled release of Ca^2+^ from the SR causes disturbed Ca^2+^ oscillations and leads to mitochondrial Ca^2+^ overload during the early phase of reperfusion [12]. Elevated Ca^2+^ concentrations promote mPTP opening, while at the same time, its mitochondrial overload enhances mtROS production and increases the pore’s sensitivity to Ca^2+^ (Figure 2). This process predominantly reflects changes in respiratory chain function, where disrupted electron flow - especially at complexes I and III-results in increased electron leak and the formation of the superoxide anion (O_2_^−^) and other mtROS [17]. In addition to Ca^2+^, there are other inducers of mPTP opening, such as low adenosine diphosphate levels, loss of the proton gradient across the IMM, increased ROS production, inorganic phosphate and adenine nucleotides, while an acidic environment (pH < 7) and Mg^2+^ keep it closed [17].

In conclusion, mPTP opening occurs during the early phase of reperfusion and its opening is predominantly influenced by Ca^2+^ entry into the mitochondria. It is important to emphasize that an increase in cytosolic Ca^2+^ alone is not sufficient to activate the pore; Ca^2+^ must be taken up into the mitochondrial matrix and its ability to trigger the mPTP opening depends on the presence of ROS, reduced ΔΨ_m_ and high phosphate concentrations. Therefore, mPTP activation is a multifactorial process that requires the simultaneous presence of these specific mitochondrial conditions [17].

### 3.3. Generation of Reactive Oxygen Species

Studies of ROS and their effects on human, animal and bacterial cells began as early as the late 19th century. At that time, the hypothesis was proposed that molecular oxygen itself could be responsible for ROS generation in bacteria and that its toxicity is further increased in the absence of the enzyme catalase. Catalase is a key enzyme in the breakdown of ROS, primarily hydrogen peroxide (H_2_O_2_), which it decomposes into water and oxygen, thereby mitigating the harmful effects of oxidative stress. In addition to catalase, superoxide dismutase (SOD) plays an important role; this enzyme eliminates another significant ROS—O_2_^−^. Superoxide dismutase converts O_2_^−^ into H_2_O_2_, which is subsequently detoxified by catalase or other peroxidases. Accordingly, it has been shown that reduced SOD expression worsens damage caused by I/R stress [25] while a cardioprotective effect was achieved by catalase expression in mitochondria [26,27].

The key question arises: what are the main sources of ROS and what are the most important targets of oxidative stress in I/R injury? Even when the discussion is limited solely to CMs, the origin and effects of ROS remain a subject of debate and there is still no definitive answer. Among the intracellular structures most frequently cited as sources of ROS, particular attention is directed to mitochondria, especially during the reperfusion phase [28]. Mitochondria contain enzymes that physiologically generate ROS and have been shown to contribute to I/R injury, among which are monoamine oxidase (MAO) and p66Shc. Monoamine oxidase produces not only H_2_O_2_, but also ammonia and aldehydes, which further disrupt the integrity of CMs. Aldehydes can be equally and sometimes more toxic than ROS themselves, whereas on the other hand, activation of aldehyde dehydrogenase 2 (ALDH2) is associated with a reduction in I/R injury. Therefore, inhibition of MAO or reduction in p66Shc expression has been shown to provide cardioprotection in experimental models. Although some studies have shown that their combined inhibition does not provide additional protective effect, MAO inhibition appears to be more significant, as MAO can be selectively inhibited by drugs that are already clinically available [27].

It is important to emphasize that the generation of mtROS can also be stimulated by processes that originate in the cytosol, such as the activation of NADPH oxidase (NOX) enzymes and the increase in intracellular Ca^2+^ concentration (Figure 2). Reactive oxygen species generated by the action of NOX can act as an initial signal that further enhances mtROS production [29] while the increased entry of Ca^2+^ into the mitochondria—although the mechanism of this is still not fully clarified—further increases their oxidative activity. At the same time, Ca^2+^ in the cytosol also activates a number of enzymes that indirectly contribute to mitochondrial stress. For example, phospholipase A_2_ releases arachidonic acid, which can modulate mitochondrial function, while the Ca^2+^—dependent calpain influences the formation of mtROS and the oxidation of proapoptotic proteins, which may contribute to their translocation into the nucleus and the activation of cell death [27,28,30].

To clarify the role of mtROS independently of cytosolic signaling, MitoParaquat was used to generate O_2_^−^ directly within the mitochondria. Findings demonstrate a dose-dependent effect: while high concentrations trigger mPTP opening, mitochondrial dysfunction and cell death, very low concentrations exert a protective effect against I/R injury. This protection, abolished by antioxidants, supports the concept of mitohormesis - the principle that low levels of mtROS can activate protective mechanisms, whereas higher concentrations induce damage [31]. Taken together, these findings indicate that mtROS can be both a consequence of cytosolic signals and active mediators of damage or protection in CMs, with the ultimate outcome depending on the intensity of oxidative stress [27].

Based on the identified mechanisms of I/R injury, specific therapeutic strategies have been developed to selectively reduce mtROS, control cytosolic Ca^2+^ overload and prevent the opening of the mPTP. In experimental models, mitoquinone (MitoQ) acts within the mitochondria to neutralize ROS and preserve respiratory chain function, while selective inhibition of complexes I and II (e.g., using amobarbital or rotenone) further limits oxidative stress [32]. The regulation of Ca^2+^ homeostasis involves the inhibition of NHE (cariporide; [33]) and the administration of sodium-glucose cotransporter 2 inhibitors (SGLT2i) (e.g., dapagliflozin [34]), which modulate ion dynamics. Similarly, targeted inhibition of NCX using compounds like SEA0400 or ORM-10962 prevents Ca^2+^ overload and contributes to the prevention of post-ischemic arrhythmias [27,35]. Furthermore, direct blockade of the MCU channel (e.g., via Ru360 [36]) reduces mitochondrial Ca^2+^ accumulation, a primary trigger for mPTP activation. Consequently, pharmacological inhibition of mPTP opening (e.g., with cyclosporine A (CsA)) has been shown to prevent the loss of cell viability [37]. These approaches collectively demonstrate that coordinated control of ROS, Ca^2+^ dynamics and mPTP stability is essential for preserving cardiac function after I/R injury.

#### Reactive Oxygen Species Generation Mediated by Succinate

Succinate, a product of the TCA cycle, accumulates significantly during tissue ischemia and is considered an important marker of ischemic stress [38]. Its accumulation occurs not only in MI but also in other conditions involving I/R injury, such as stroke, organ transplantation, resuscitation, physical exertion and inflammation. The reason for succinate accumulation during ischemia is the lack of oxygen and low pH, which leads to the reduction in coenzyme Q (CoQ) and consequently the formation of large amounts of succinate. During ischemia, succinate dehydrogenase (SDH) functions in reverse, reducing fumarate to succinate [39]. The primary source of fumarate is the breakdown of adenosine monophosphate through the purine nucleotide cycle. Fumarate is considered to be primarily converted into malate because this way it is more easily transported into the mitochondria via the dicarboxylate carrier [40]. This exchange of succinate and malate between the mitochondria and the cytosol further maintains elevated levels of succinate in the tissue. During reperfusion, succinate is rapidly oxidized via SDH and with reduced CoQ, the so-called reverse electron transport (RET) occurs, which is responsible for generating the largest amount of ROS, predominantly O_2_^−^, at complex I. Its existence has been demonstrated in in vitro and in vivo models and thus RET has been identified as the primary source of mtROS [27] (Figure 2).

Targeting this mechanism provides significant protection against I/R injury; for example, inhibition of complex I or RET (e.g., with rotenone or mitochondria-targeted S-nitrosothiol (MitoSNO)) reduces damage, but it should not be overlooked that prolonged inhibition of complex I can impair bioenergetics and cause cardiomyopathy [41]. A more effective approach is the modulation of succinate metabolism through SDH inhibition, e.g., with malonate, which prevents succinate accumulation and its oxidation during reperfusion, reducing RET and ROS production [42].

These findings highlight the key role of succinate in I/R injury and confirm that the regulation of its metabolism, particularly through SDH inhibition, represents a promising strategy for cardioprotection. Controlling succinate accumulation and RET-induced ROS can significantly reduce oxidative stress and tissue damage [27].

## 4. Regulation of Cellular and Mitochondrial Mechanisms During Ischemia-Reperfusion Injury

### 4.1. Regulation of Mitochondrial Permeability Transition Pore Opening

At the center of the molecular regulation of mPTP opening is cyclophilin D (Cyp-D), so far, the only experimentally confirmed essential regulatory component of the mPTP. By composition, it is a protein soluble in the mitochondrial matrix and a key inducer of mPTP opening. Cyclophilin D modulates the pore’s sensitivity to Ca^2+^, phosphate and ROS through interactions with candidate structural elements (ANT, PiC, ATP synthase), thereby significantly lowering the threshold for pathological opening. Genetic models consistently confirm that Cyp-D deletion significantly reduces the likelihood of sustained opening, while its overexpression increases mitochondrial susceptibility to Ca^2+^-induced permeabilization [17,43].

Based on these findings, among the most significant and well-studied approaches aimed at pharmacological modulation of Cyp-D, CsA stands out. This inhibitor of the regulatory component Cyp-D has shown a cardioprotective effect in phase 2 studies in patients with ST-elevation myocardial infarction (STEMI). However, larger randomized trials have not confirmed these findings, showing neutral or negative effects on infarct size and clinical outcomes [37].

Three key factors may explain this discrepancy:

1. Pharmacokinetics and drug delivery: mPTP opens within the first few minutes of reperfusion, making the early period critical for effective pharmacological intervention. Cyclosporin A acts by inhibiting Cyp-D, the regulatory component of the mPTP, but its distribution to the mitochondria is not immediate. Studies have shown that CsA reaches mitochondria slowly and the different formulations used in clinical trials may further delay achieving an effective concentration in target tissues. These delays are particularly significant because the mPTP opening occurs immediately after reperfusion, so the drug must be present in the mitochondria during this critical time window to exert a protective effect. Due to these pharmacokinetic limitations, even a drug that is effective in preclinical models may show neutral or negative results in large clinical trials [44].
2. Drug specificity: CsA acts on Cyp-D, the pore regulator and not on the pore itself, while in some experimental models, cardioprotective effects independent of the mPTP have also been observed [45].
3. Patient selection and heterogeneity: In large clinical trials, patients with significantly heterogeneous clinical profiles, varying ischemia durations, comorbidities, ages and sexes were included - all factors that subsequent analyses show can influence the response to therapy independently of infarct size. Preclinical models, in contrast, typically involve young, healthy animals without comorbidities, with strictly controlled occlusion and reperfusion times, which significantly limits translation. Additionally, there is evidence that various comorbidities (e.g., diabetes, hypertension, renal dysfunction) and sex can modulate clinical outcomes and the potential response to mPTP inhibitors, although available studies have not yet provided sufficient data to clearly quantify these differences [46].

Pharmacological inhibition of Cyp-D, particularly with CsA and its derivatives, has shown strong cardioprotective effects in preclinical settings, but their translation into clinical practice remains limited. This gap between preclinical efficacy and clinical failure likely arises from a combination of complex reperfusion signaling networks, limited CsA specificity, pharmacokinetic challenges and patient heterogeneity, including sex and comorbidities. Additionally, the fact that Cyp-D inhibition predominantly affects lethal, but not physiological transient mPTP activity further limits the clinical potential of such approaches [46].

Together, these factors highlight the need for more precisely targeted therapies and careful design of future cardioprotective strategies.

### 4.2. Regulation of Enzymatic Activity During Myocardial Ischemic Injury

Although the increase in intracellular Ca^2+^ plays a key role in I/R injury and participates in the activation of numerous enzymes, their regulation does not depend exclusively on the concentration of this ion. Enzymatic activity is also modulated by additional factors, such as pH, the degree of phosphorylation, subcellular localization and interactions with endogenous inhibitors [30].

One example of the complex regulation of enzymes during ischemia is calpain, an intracellular cysteine protease activated by Ca^2+^. Although increased Ca^2+^ concentration can contribute to its activation, it is not sufficient by itself during ischemia, as cytoplasmic acidification significantly inhibits calpain activation. Its activity reaches a maximum only during reperfusion, when pH returns toward normal values, while additional factors, such as interaction with the endogenous inhibitor calpastatin and subcellular localization, also modulate enzyme activation. This example illustrates that protease activation does not depend solely on Ca^2+^ and that enzyme regulation during ischemia is multilayered and pH-dependent [30,47].

Regarding phospholipases, this is a heterogeneous group of enzymes that includes both Ca^2+^-dependent and Ca^2+^-independent isoforms. Their activity does not depend exclusively on Ca^2+^ concentration, but also on other factors, such as pH, phosphorylation and the state of the cell membrane. For example, cytosolic phospholipase A_2_ isoforms are activated by Ca^2+^ binding and phosphorylation, and their optimal activity is achieved at physiological pH. Calcium-independent phospholipase A_2_, particularly the β isoform (iPLA_2_β), is present in the myocardium and mitochondria, where it is catalytically active and associated with the inner mitochondrial membrane. It has been experimentally shown that its activity increases during I/R, leading to the degradation of mitochondrial phospholipids, release of arachidonic acid and CMs damage. Inhibition of iPLA_2_β using bromoenol lactone reduces infarct size and provides cardioprotection, confirming the harmful role of excessive activity of this phospholipase [48].

Based on these data, it can be concluded that enzymatic activity during ischemia does not depend solely on Ca^2+^ overload, but is also modulated by other factors, such as pH, phosphorylation, intracellular enzyme localization and membrane state.

## 5. Activation of Inflammatory Pathways and the Immune System

During myocardial I/R injury, stressed CMs release various endogenous molecules known as damage-associated molecular patterns (DAMPs), which activate the innate immune system and contribute to inflammation in cardiac tissue. These molecules are detected through pattern recognition receptors, particularly Toll-like receptors, predominantly TLR2 and TLR4, which are expressed in cardiac tissue [8,49].

Among the most studied DAMP molecules are Heat Shock Proteins (HSPs), including HSP60, HSP72 and HSC70, which can be passively released from necrotic cells, but also actively secreted via exosomes or lipid-raft-dependent mechanisms [50,51]. Extracellular HSP60 and HSP72 activate TLR2 and TLR4 in immune cells, while HSP60 can also activate TLR4 in CMs themselves, triggering MyD88-dependent signaling pathways and inducing inflammatory responses [52]. Besides HSPs, another significant DAMP in the myocardium is HMGB1, a nuclear DNA-binding protein, which is released passively during necrosis or actively by stressed CMs and can activate TLR2, TLR4 and TLR9 [53]. Extracellular HMGB1 is associated with increased inflammation and larger infarct size; however, its nuclear fraction is essential for genome stability, and its post-ischemic release may stimulate limited reparative responses through progenitor cell activation [51]. The release of DAMP molecules during I/R represents a key mechanism for initiating the immune response in injured myocardium, but it also contributes to the progression of inflammation and functional decline of the heart [51].

After activation of TLR receptors by DAMPs, signaling pathways proceed through TRIF- and MyD88-dependent mechanisms, resulting in the activation of transcription factors interferon regulatory factor 3 and nuclear factor kappa B and consequently the synthesis of pro-inflammatory cytokines such as interleukin-1 (IL-1), interleukin-6 (IL-6) and tumor necrosis factor-alpha (TNF-α). These cytokines promote the recruitment and activation of neutrophils at the site of myocardial injury, thereby initiating the inflammatory response [8,54]. Damage-associated molecular patterns and inflammatory mediators can also contribute to platelet activation and migration. In this way, the interaction between leukocytes and platelets can induce the production of various mediators, including chemotactic molecules, adhesion molecules, released proteins, diverse pro-inflammatory lipids and chemokines. In the first step, P-selectin on the activated platelet interacts with its receptor, P-selectin glycoprotein 1 on leukocytes, inducing a signaling pathway that enables stable leukocyte-endothelial interactions and contributes to the progression of I/R injury [1,8].

Conversely, the adaptive immune system, composed of T and B lymphocytes, not only contributes to myocardial damage caused by I/R injury but also plays a key role in reparative processes and tissue regeneration [55]. B cells play a key role in adaptive immune responses as they produce thousands of antibodies upon activation [8,56]. Earlier experimental studies have shown that IgM is responsible for initiating inflammatory responses in the myocardium through complement activation. In this way, blocking IgM can reduce complement activation and alleviate disease severity. However, there is still limited data on the role of B cells in the pathogenesis of myocardial injury.

On the other hand, CD4^+^ T lymphocytes should not be overlooked, as they exacerbate myocardial injury through the production of pro-inflammatory cytokines, such as interferon-γ and TNF-α [8,57]. In contrast, CD4^+^CD25^+^ regulatory T cells (Tregs), a specific subpopulation of CD4^+^ T lymphocytes, exhibit a protective effect in the context of I/R injury. Experimental models have shown that depletion of Treg cells leads to more pronounced myocardial damage and increased infiltration of inflammatory cells, whereas their adoptive transfer attenuates the inflammatory response and reduces the extent of injury [8,58]. Treg cells exert their immunomodulatory function through contact-dependent mechanisms as well as by secreting anti-inflammatory cytokines, including interleukin-10 and transforming growth factor-beta [58].

### The Role of the Spleen in Immunoregulation and Cardioprotection After I/R Injury

The spleen is a central immune organ and a key link between the immune system, the autonomic nervous system and the circulation. It has both a regulatory and a pathological role in ischemic heart disease and myocardial remodeling after infarction [59]. It serves as a key reservoir and a maturation site for specific immune cell populations; however, under pathological conditions, such as chronic inflammation or ischaemic stress, it may also become a site for extramedullary haematopoiesis [60,61].

The spleen, although primarily an immunological organ, has emerged as an important modulator of cardiovascular processes, particularly in the development and progression of atherosclerosis. Under conditions of hyperlipidaemia, experimental studies, predominantly in mice and rats, show that its activation leads to the enhanced production and mobilization of leukocytes, which intensifies inflammation within the vascular walls. Preclinical models demonstrate that alterations in splenic endothelial cells promote the release of inflammatory cells, whereas the removal or denervation of the spleen reduces atherosclerotic progression and increases plaque stability, confirming its active and potentially detrimental role [61].

For instance, macrophages that clear oxidized low-density lipoprotein (LDL) in the early stages transition into a proinflammatory phenotype in later stages, releasing cytokines and proteases that weaken the plaque structure. Specific sub-populations of B and T cells further exacerbate inflammation and disrupt immunological balance. In both experimental murine models and clinical observations following MI, the spleen is further activated, enhancing the production of myeloid cells and accelerating the infiltration of inflammatory cells into atherosclerotic lesions, thereby worsening the disease course. These findings indicate that the spleen represents a significant source of inflammatory signals that contribute to the progression and instability of the atherosclerotic plaque. However, alongside these pro-inflammatory and potentially harmful effects, the spleen also possesses significant mechanisms of cardioprotection following I/R injury [61].

The most significant cardioprotective effect of the spleen after I/R injury is achieved via the vagus-splenic axis as follows: activation of efferent vagal and noradrenergic splenic nerves stimulates β2-adrenergic receptors on splenic T cells. This leads to the release of acetylcholine and activation of α7-nicotinic receptors on macrophages, thereby reducing the inflammatory response [62]. Preclinical studies demonstrate that the vagus-splenic axis enables cardioprotection also through auricular tragus stimulation and remote ischemic conditioning (RIC) in murine and porcine models, as well as in clinical studies involving humans [63].

Epidemiological data also indicate a protective role of the spleen, with splenectomy being associated with a higher risk of MI, stroke and long-term mortality from ischemic heart disease [61]. In experimental settings splenectomy before MI did not reduce infarct size in pigs after 60 min of occlusion and 3 h of reperfusion [64], whereas in mice with permanent coronary artery ligation, it reduced infarct size after 24 h of reperfusion, indicating that the effect of the spleen depends on the specific animal model and the duration of reperfusion [61,65].

It should not be overlooked that certain pharmacological modulators, such as aspirin, indomethacin and P2Y_12_ antagonists, can modulate the splenic immune response and myocardial inflammation [66], while anesthetics such as atropine and propofol can abolish the cardioprotective effect of RIC [67]. The molecular mechanisms of these effects are not yet fully understood, so this area remains the subject of active research.

Thus, the spleen functions as a central regulator connecting the immune, nervous and vascular systems. For this reason, it is important not to overlook it as an organ that plays a key role in I/R injury while also representing a potential target for the development of new therapeutic strategies.

## 6. The Role of Excessive Cardiomyocyte Contraction in Acute Myocardial Infarction (Mechanisms and Therapeutic Approaches)

Hypercontraction of CMs represents a key mechanism of myocardial injury during acute infarction and the two main types are Ca^2+^ overload-induced contraction and rigor-type contraction [68].

Calcium overload-induced contraction occurs when ATP production in CMs resumes after a period of Ca^2+^ accumulation during ischemia. In this way, a high level of cytosolic Ca^2+^ in the presence of ATP triggers uncontrolled contraction of the myofibrils [69]. During reperfusion, the sudden return of oxygen enables the restoration of oxidative phosphorylation and ATP resynthesis, which reactivates actin-myosin interactions faster than the excess Ca^2+^ can be removed, leading to hypercontraction. Additionally, cellular acidification during ischemia causes Na^+^ overload via the NHE, reducing Na^+^/K^+^-ATPase activity, while the SR and NCX contribute to further Ca^2+^ accumulation [70]. Calcium overload-induced hypercontraction is also associated with mPTP opening, leading to mitochondrial swelling, dysfunction of the electron transport chain complexes, release of Cyt c and activation of apoptotic pathways [71].

On the other hand, rigor-type contraction occurs under conditions of low ATP availability and is not initiated by Ca^2+^ overload. During reoxygenation, prolonged ATP depletion allows slow cross-bridge cycling between actin and myosin, leading to sustained contraction and the formation of rigor bonds [72]. Rigor contraction causes less pronounced cell shortening compared to Ca^2+^-induced contraction, but it can worsen CMs injury through prolonged energy depletion and mPTP opening, leading to necrosis. Overall, both mechanisms demonstrate how hypercontraction represents a significant factor in the pathophysiology of acute MI, with differences in phenotype and underlying causes [71].

Given this, therapeutic strategies are aimed at reducing hypercontraction and preserving cellular integrity. β-blockers (metoprolol, propranolol, landiolol) reduce contraction-induced injury by modulating Ca^2+^ homeostasis [71]. Experimentally, Ca^2+^ influx inhibitors, such as the NCX inhibitor KB-R7943, are also effective, preventing Ca^2+^ overload and reducing hypercontraction during reperfusion [73]. The clinical effects of Ca^2+^ channel blockers are mixed: in patients with preserved left ventricular ejection fraction, they may be protective, whereas non-dihydropyridine blockers can increase the risk of heart failure [71].

The latest strategy involves selective myosin inhibitors, mavacamten and aficamten, which reduce the formation of actin-myosin cross-bridges in sarcomeres, limit hypercontraction and decrease infarct size in in vivo experimental models, while preserving hemodynamics [74]. These drugs have also been confirmed in vitro, where they prevent excessive shortening of CMs and preserve cellular integrity [71].

In conclusion, therapeutic strategies against hypercontraction combine the control of contractility and Ca^2+^ homeostasis. β-blockers are currently the most well-studied and clinically applicable, whereas myosin inhibitors and Ca^2+^ blockers represent promising experimental approaches that require further clinical investigation.

## 7. Cellular Forms of Death During Ischemia/Reperfusion Injury

**Figure 3:**
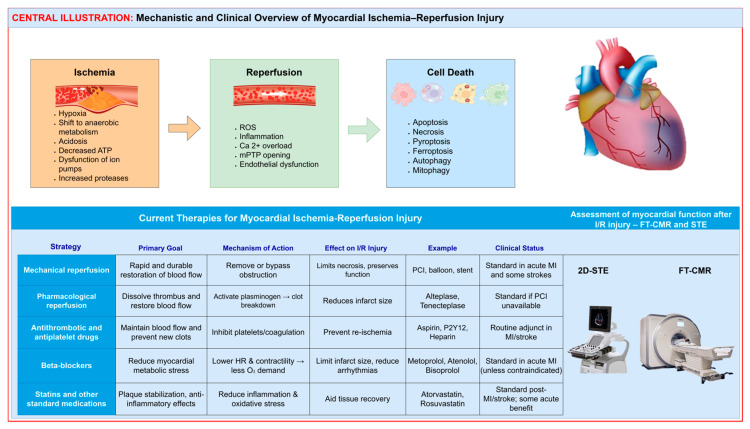
Mechanistic and clinical overview of myocardial ischemia-reperfusion injury. The diagram illustrates key pathophysiological events, current therapeutic approaches, and the assessment of myocardial function. Abbreviations: 2D-STE, two-dimensional speckle tracking echocardiography; ATP, adenosine triphosphate; FT-CMR, feature tracking cardiac magnetic resonance; mPTP, mitochondrial permeability transition pore; PCI, percutaneous coronary intervention; ROS, reactive oxygen species.

Cell death represents a key event in myocardial I/R injury, determining infarct size and functional recovery of the heart. Traditionally, it has been classified into two types: apoptosis and necrosis. Apoptosis is known as programmed cell death that occurs under the control of precise molecular pathways, whereas necrosis was previously considered uncontrolled death resulting from acute injury. However, recent research reveals that there are also forms of necrosis that are regulated, such as necroptosis, ferroptosis and pyroptosis, which are now classified as “programmed necrosis,” i.e., programmed cell death [75,76] (Figure 3).

### 7.1. Apoptosis

Apoptosis, a form of programmed cell death, plays a key role in CMs injury during I/R injury. This is particularly relevant because CMs rely heavily on mitochondrial ATP production; indeed, more than 90% of cellular ATP is generated by mitochondria, so any mitochondrial dysfunction directly impairs their viability [77]. During ischemia, reduced oxygen supply causes hypoxia, leading to the death of a significant number of CMs, while reperfusion, although necessary to restore cellular metabolism, increases CMs death [78].

Apoptosis in CMs is activated through two main pathways. The intrinsic, also known as the mitochondrial pathway, is triggered by increased mitochondrial membrane permeability, allowing the release of pro-apoptotic proteins such as Cyt c and high-temperature requirement protein A2. The released Cyt c then binds to apoptotic protease-activating factor 1 and caspase-9, forming a complex that activates caspase-3 and initiates programmed cell death [79]. The regulatory proteins Bax and Bcl-2 play a key role in this process: while Bax promotes outer mitochondrial membrane permeability, Bcl-2 exerts anti-apoptotic effects, and their ratio determines the intensity of apoptosis [80]. The extrinsic pathway, on the other hand, is activated through death receptors or pattern-recognition receptors such as TLR4, which also leads to caspase activation and cell death. Its activity can be modulated by regulatory proteins and specific miRNAs that directly affect caspases [81].

During ischemia itself, apoptosis occurs relatively gradually. Hypoxia reduces ATP availability and activates intrinsic death mechanisms, while mitochondrial fission increases to isolate damaged fragments and preserve the functionality of the remaining mitochondria. The release of pro-apoptotic proteins during this period is limited and selective mitophagy removes damaged mitochondria, thereby mitigating CMs death. Autophagy and mitophagy act as protective mechanisms, maintaining cellular homeostasis and reducing stress during ischemia [77,82].

During the reperfusion phase, the dynamics of apoptosis change significantly. Specifically, the sudden oxygen influx and elevated intracellular Ca^2+^ levels trigger massive mPTP opening and IMM rupture (Figure 2). This allows rapid release of Cyt c and other pro-apoptotic proteins into the cytosol, accelerating the activation of caspase-9 and caspase-3 and intensifying cell death [79]. At the same time, mitochondrial fission continues, while excess ROS further amplifies apoptosis. Although mitophagy is still active, its protective effects are insufficient to prevent massive cell death [77].

It is clear that the behavior of apoptosis differs between ischemia and reperfusion. During ischemia, the process is relatively controlled, with limited release of pro-apoptotic factors and active protection through autophagy and mitophagy [83], whereas reperfusion leads to rapid and massive activation of apoptotic pathways, causing significant loss of functional CMs and worsening myocardial injury [78]. These events highlight the complexity of cellular survival and death processes, with direct consequences for the heart’s ability to recover after I/R injury.

### 7.2. Necroptosis

Necroptosis represents a significant mechanism of CMs loss in myocardial I/R injury. Several molecules, including cytokines from the TNF family and TLR receptors, can trigger this form of programmed necrosis. Activation of the receptor-interacting protein kinase 1 (RIPK1)-receptor-interacting protein kinase 3 (RIPK3)-mixed-lineage kinase domain-like pseudokinase (MLKL) signaling cascade represents the key pathway leading to cell membrane disruption and lysis during necroptosis [8].

Rat models of myocardial I/R injury, as well as cellular hypoxia/reoxygenation models, have shown a significant increase in TNFα, RIPK1, RIPK3 and p-MLKL, which sustains activation of the necroptotic pathway. Inhibition of this pathway using resveratrol has been shown to reduce the number of necroptotic cells and infarct size [75,84]. However, interpreting these findings requires caution. Pharmacological necroptosis inhibitors, such as necrostatin-1, also possess off-target effects, including action on the enzyme indoleamine 2,3-dioxygenase, which can affect the validity of experimental conclusions. Additionally, most experimental data come from rodent models; data from human myocardium are limited and existing studies suggest that the effects of necroptosis and the efficacy of its inhibitors in humans may be lower than observed in animal models [85,86].

It has been shown that the RIPK3-phosphoglycerate mutase family member 5 (PGAM5)-dynamin-related protein 1 (Drp1) signaling pathway is also activated during I/R injury. Phosphoglycerate mutase 5, a mitochondrial membrane protein and Ser/Thr phosphatase, is phosphorylated by RIPK3, leading to the activation of Drp1 independently of its phosphatase activity, which induces mitochondrial fragmentation and necroptosis [75,87]. Pharmacological inhibition or genetic deletion of PGAM5 can protect the heart by reducing necroptosis through enhanced mitochondrial quality control [75,88,89].

However, it should be emphasized that the role of PGAM5 and necroptosis itself is not straightforward: in contrast to earlier hypotheses, studies in PGAM5-deficient mice have shown that the absence of PGAM5 worsens myocardial injury after I/R, as PGAM5 also functions as an important regulator of mitophagy and mitochondrial homeostasis, which protects against necroptosis [90]. This suggests that the population of injured CMs may depend on the balance between necroptosis activation and the cells’ ability to eliminate damaged mitochondria.

Moreover, although some studies have reported that 4-hydroxy-2-nonenal (4-HNE) can induce necroptosis, it is important to note that 4-HNE is a product of lipid peroxidation that can also trigger other cell death pathways, including oxidative stress-mediated death or even ferroptosis [91]. Therefore, it cannot be stated with certainty that 4-HNE-dependent cell death in the heart after I/R must be necroptosis; alternative pathways remain possible and warrant further investigation.

A similar involvement has been demonstrated for the phosphoinositide 3-kinase/protein kinase B (PI3K/Akt) pathway, which is downregulated following I/R injury due to decreased expression of the glucagon-like peptide-1 receptor (GLP-1R). Treatment with GLP-1R agonists restores GLP-1R/PI3K/Akt pathway activity and inhibits necroptosis by suppressing p-RIPK3 and p-MLKL [92].

Furthermore, studies in mouse models and in vitro I/R models have shown that increased RIPK3 expression can induce endoplasmic reticulum stress and intracellular Ca^2+^ overload, leading to the activation of xanthine oxidase (XO). Excessive production of ROS via XO mediates mPTP opening and necroptosis in CMs, while ER stress inhibitors have the potential to improve cardiac function following I/R injury [75,93] (Figure 2).

Finally, the transient receptor potential canonical 6 channel, associated with Ca^2+^ regulation, is highly expressed during myocardial I/R injury. Its activation causes intracellular Ca^2+^ overload and the increased concentration of this ion triggers Ca^2+^ /calmodulin-dependent protein kinase II (CaMKII) phosphorylation, which promotes mPTP opening and contributes to necroptosis [75,94].

In addition to apoptosis and necrosis, other, less well-known forms of regulated cell death also participate in ischemic injury, including autophagy-mediated cell death (autophagy), inflammation-mediated cell death (pyroptosis) and iron-mediated cell death (ferroptosis) [1].

### 7.3. Pyroptosis

Pyroptosis is a pro-inflammatory form of cell death that is activated in response to signals recognized by cytosolic receptors pathogen-associated molecular patterns (PAMPs) or DAMPs [1,4,95]. Histologically, pyroptosis is characterized by cytoplasmic swelling, formation of pyroptotic bodies, plasma membrane rupture and release of cellular contents, while mitochondrial integrity is largely preserved [96].

The pyroptotic inflammasome activates pro-caspase-1, which leads to the release of the inflammatory cytokines IL-1β and IL-18 and to the processing of gasdermin D (GSDMD) into its active form. The active GSDMD then participates in the formation of pores in the plasma membrane through homopolymerization, allowing membrane perforation and cell lysis, thereby triggering a strong local inflammatory response [1,97].

In the myocardium, pyroptotic pathways can be divided into canonical and non-canonical. The canonical pathway involves activation of the NLR family pyrin domain containing 3 inflammasome and caspase-1. Studies have shown that deletion of the pro-caspase-1 gene reduces infarct size in mice in an I/R injury model and alleviates the phenotype clearly indicating the contribution of the canonical pyroptosis pathway to infarct expansion [1,98].

The noncanonical pathway of pyroptosis in rodents involves caspase-11, while in humans it involves caspases-4/5. The caspase activation and recruitment domain (CARD) functions as a sensor for lipopolysaccharide (LPS). The binding of LPS to CARD induces oligomerization and activation of caspase-4/5/11. Active caspases-4/5/11 directly cleave GSDMD, which leads to cell lysis, while they do not cleave pro-IL-1β or pro-IL-18. Through the activation of the caspase-1 inflammasome, caspases-4/5/11 indirectly enable the maturation and release of IL-1β and IL-18, thereby linking with the canonical inflammatory effect [1,75,98].

These pathways show that pyroptosis in the myocardium functions in close association with apoptotic and necroptotic mechanisms. Although pyroptosis contributes to progressive inflammation, microvascular dysfunction and potential infarct size expansion, precisely quantifying its relative contribution compared to necrosis or necroptosis remains uncertain. Given the complexity of the interconnected pathways, it is likely that pyroptosis acts as an amplifier of the inflammatory response and a mediator in infarct progression, but the overall infarct size results from the combined action of multiple forms of cell death [99].

Therapeutically, inhibition of GSDMD pores or blockade of proteases associated with the pyroptosome has shown protective effects in animal models of I/R injury, indicating potential targeted modulation of pyroptosis to reduce infarct size and inflammation. Nevertheless, future research is needed to determine the roles of canonical versus non-canonical pathways and to better understand the interdependence of pyroptosis with other forms of cell death in the myocardium [99].

### 7.4. Ferroptosis

Ferroptosis is a non-apoptotic form of programmed cell death that arises as a consequence of combined depletion of antioxidant reserves and disruption of iron metabolism during the early phases of reperfusion. During this phase, the sudden increase in oxygen and ROS under conditions of depleted glutathione (GSH) leads to destabilization of membrane phospholipids rich in polyunsaturated fatty acids. A key regulator of this process is glutathione peroxidase 4 (GPX4), whose loss of activity during the first minutes to hours of reperfusion allows uncontrolled accumulation of lipid hydroperoxides and initiates the ferroptosis cascade, with secondary mitochondrial damage [100].

In addition, increased intracellular Fe^2+^—whether due to enhanced uptake via transferrin receptor 1, release of iron from ferritin, or mitochondrial dysfunction - further promotes the Fenton reaction and exacerbates membrane damage [101]. These mechanistic points form the basis for the most promising therapeutic approaches targeting ferroptosis.

Pharmacological preservation of GPX4 or stabilization of GSH reserves has been shown to be most effective when applied in the very early reperfusion period, before lipid peroxidation accelerates irreversibly [102]. Similarly, iron chelation shows a protective effect only when performed during the phase of active free Fe^2+^ accumulation, that is, before phospholipid peroxidation reaches the threshold leading to membrane collapse [103].

Lipophilic radical scavengers, such as ferrostatin-1 and liproxstatin-1, act by interrupting the chain reaction of lipid peroxidation and are therefore therapeutically most effective within a few hours after reperfusion, while ROS-mediated damage predominates and before the secondary inflammatory cascade occurs [104].

Together, these approaches indicate that ferroptosis is pharmacologically highly time-sensitive: effective modulation of this pathway requires early intervention, when the imbalance between GPX4 activity, available iron and lipid radicals is just beginning to develop and before the process progresses to a self-propagating state.

## 8. Autophagy and Mitophagy-Adaptive or Harmful Responses

Although apoptosis, necrosis, pyroptosis and ferroptosis are forms of cell death—with the first, pyroptosis and ferroptosis being regulated—cells also possess adaptive mechanisms that primarily serve for survival. Among these, autophagy and mitophagy represent key intracellular pathways that maintain homeostasis by removing damaged proteins and organelles, recycling nutrients and reducing oxidative stress. Although primarily protective, these processes can, under conditions of excessive activation or imbalance, contribute to a form of regulated cell death dependent on autophagy (autophagy-dependent cell death) [1,4,8,9].

### 8.1. Autophagy

Autophagy is an intracellular survival mechanism that enables the degradation and renewal of damaged proteins and organelles within lysosomes it is one of the key mechanisms for maintaining myocardial homeostasis under stress conditions, limiting cardiac damage and preserving its function. Although generally protective, maladaptive autophagy—whether insufficient or excessive—can contribute to disease progression [1].

To date, three types of autophagy have been described: macroautophagy, microautophagy and chaperone-mediated autophagy. However, today the most studied process is macroautophagy, which occurs through three key steps: formation of the phagophore, maturation of the autophagosome and fusion with lysosomes to form the autolysosome [105]. Its main regulators include mechanistic target of rapamycin (mTOR) as an inhibitor and AMP-activated protein kinase (AMPK) and glycogen synthase kinase 3 beta (GSK-3β) as positive modulators [106].

Autophagy changes significantly during the phases of ischemia and reperfusion. During ischemia, its role is enhanced, especially in partially damaged CMs in the border zone of the infarct. This increase in autophagy helps restore damaged mitochondria and reduce cellular injury and it is initiated by the activation of the previously mentioned p-AMPK, GSK-3β and oxidative stress-responsive enzymes, including SOD1, ALDH2 and NOX4. Mitochondrial enzymes and ubiquitin-proteasome pathways, such as Parkin-mediated mitophagy, further contribute to the protection of CMs [105].

On the other hand, the role of autophagy in reperfusion injury is more complex. Reperfusion, although necessary for the restoration of blood flow, can reverse the role of autophagy from protective to harmful. Early reperfusion further stimulates autophagy and the autophagic flux, which can trigger autosis, a type II form of programmed cell death dependent on autophagy. In this context, key roles are played by molecules such as Beclin 1 and Run domain Beclin 1-interacting and cysteine-rich domain-containing protein, whose interaction determines the transition of autophagy from an adaptive to a pathological phase. Excessive phagophore formation or inadequate autophagosome maturation leads to the accumulation of vacuoles and cellular damage. Interventions targeting the early phase of autophagosome formation, such as class III phosphoinositide 3-kinase inhibitors, have shown promising results in reducing autosis [105].

In conclusion, autophagy represents a mechanism that can play a dual role within the cell. Its excessive activation can lead to self-digestion and cell death, while insufficient autophagy—caused, for example, by apoptotic or necroptotic signals or increased zinc levels in lysosomes during I/R—can prompt a potentially damaged cell to enter programmed death prematurely. The challenge lies in maintaining an optimal level of autophagy—active enough to efficiently remove damaged proteins and organelles, but not so excessive that it becomes harmful itself. As a key homeostatic process, autophagy enables the recycling of worn cellular components and contributes to cell survival, while its detrimental effect arises only when it becomes excessive, a condition known as autosis [27].

Particularly important within autophagy is mitophagy, which represents the selective degradation of damaged mitochondria. Similarly to general autophagy, its effects can be protective or pathogenic, depending on the phase of I/R injury and the degree of activation of this process [27].

### 8.2. Mitophagy

Mitophagy is a selective form of mitochondrial autophagy that is crucial for CMs survival and for limiting I/R injury.

During the ischemic phase, due to a lack of oxygen and nutrients, mitochondria lose the ability to adequately produce ATP, leading to the accumulation of damaged and nonfunctional mitochondria. At this stage, moderate activation of mitophagy acts protectively: it removes dysfunctional mitochondria, reduces ROS production and maintains the energetic and oxidative balance of cells, thereby aiding CMs survival [27,107].

However, in the reperfusion phase, when oxygen returns to the tissue, there is a sudden increase in oxidative stress, ROS generation and mPTP opening. If mitophagy is excessively active at this stage, it leads to excessive loss of mitochondria, decreased ATP production and induction of cell death, which worsens the infarction process and myocardial damage. On the other hand, insufficient mitophagy during reperfusion prevents the removal of damaged mitochondria, which also leads to the accumulation of ROS, activation of apoptosis and inflammation and worsening of I/R. Thus, the effect of mitophagy depends on the phase and intensity: moderate mitophagy during ischemia and early reperfusion has a protective effect, whereas excessive or insufficient activation during reperfusion becomes harmful [107].

The central regulators of this process are the proteins PTEN-Induced Putative Kinase 1 (PINK1) and Parkin RBR E3 Ubiquitin Protein Ligase, while the tumor suppressor protein p53 and members of the Bcl-2 family (MCL-1, Bcl-xL, Bcl-2) act as negative regulators of mitophagy [8,108]. In addition, the deubiquitinase USP30, localized in mitochondria, also inhibits Parkin-mediated mitophagy, thereby leading to cytotoxic damage and increased cell death [109]. On the other hand, ALDH2 exerts a cardioprotective effect in I/R injury, as it is essential for preserving cardiac function through the regulation of PINK1/Parkin-mediated mitophagy. Activation of ALDH2 by agonists such as Alda-1 shows potential as a therapeutic approach for myocardial I/R injury [8,110].

Therapeutically, mitophagy can be modulated by drugs or other interventions to achieve an optimal balance: certain agents, such as melatonin or T3, can block excessive mitophagy during [111], while drugs such as *Panax notoginseng* saponins or berberine can enhance protective mitophagy during the ischemic phase. Maintaining this dynamic balance between protective and harmful mitophagy is key to reducing myocardial injury and improving heart function after I/R [112].

Despite the protective roles of autophagy and mitophagy, excessive or dysregulated activation of these processes can exacerbate myocardial injury. Therefore, precise modulation of these mechanisms is crucial for the development of effective therapeutic strategies in myocardial I/R injury [1].

## 9. The No-Reflow Phenomenon and Microvascular Obstruction After Reperfusion: Their Clinical and Diagnostic Relevance

Reperfusion via percutaneous coronary intervention still represents the standard and fastest treatment for STEMI. Although the restoration of patency in epicardial coronary arteries reduces myocardial damage in a large number of patients, hypoperfusion of the myocardial tissue remains present. This phenomenon, known as no-reflow, is primarily a consequence of severe dysfunction of the microvascular system or the loss of its integrity, which leads to MVO [113].

The pathophysiology of MVO is complex and multifactorial. Clinical and experimental studies indicate that several mechanisms contribute to the development of the no-reflow effect. Among them are distal embolization of thrombotic or plaque material, active vasoconstriction mediated by sympathetic reflexes and α-adrenergic mechanisms and endothelial dysfunction induced by cytokines and humoral factors such as TNFα, serotonin, thromboxane and endothelin [114]. Additionally, platelet and platelet-leukocyte aggregates, erythrocyte stasis, compression of capillaries due to extravascular edema, as well as structural damage to the capillary endothelium, including endothelial cell detachment and capillary hemorrhages, participate in MVO [115,116]. These mechanisms of MVO directly affect diagnostic indicators, such as cardiovascular magnetic resonance (CMR) assessment of infarct size and MVO and correlate with clinical outcomes [114].

The use of CMR to assess MVO and infarct size was the primary focus of an individual patient data pooled analysis of seven randomized primary percutaneous coronary intervention (PCI) studies in patients with STEMI. This analysis created the conditions for precise measurement of MVO and infarct mass within 7 days after reperfusion and for examining their association with clinical outcomes, including all-cause mortality, hospitalization for heart failure and reinfarction. Through this methodologically robust study, CMR demonstrated its advantage not only in detecting MVO but also in quantifying infarct size, which enabled reliable patient risk stratification [114].

The results show that MVO is strongly and independently associated with increased mortality and hospitalization for heart failure during one-year follow-up, while infarct size further contributes to prognostic validity. By introducing CMR in the early phase after primary PCI, it is possible to better detect high-risk patients and provide a comprehensive assessment of myocardial status, emphasizing the importance of this method in clinical practice and future studies aimed at interventions that can reduce MVO and improve outcomes [113].

Echocardiographic assessment of myocardial deformation (“strain”) represents an auxiliary tool to CMR in evaluating myocardial damage after STEMI. Speckle-tracking 2D-strain allows quantification of longitudinal, radial and circumferential function of the left ventricle, with longitudinal strain being particularly sensitive to subendocardial necrosis, while circumferential strain helps in distinguishing subendocardial from transmural infarction. Studies show that global longitudinal strain is associated with the extent of MVO assessed by CMR in the first days after reperfusion, as well as with infarct size at 3-month follow-up, providing prognostically significant information about regional and global myocardial function. In this way, strain can complement CMR in the early identification of patients at higher risk and contribute to a more detailed depiction of the severity of myocardial damage [117] (Figure 3).

In the following paragraph, we will consider the role of pericytes in maintaining microvascular integrity and their contribution to the development of MVO after reperfusion.

### The Role of Pericytes in the Development of Microvascular Obstruction

An increasing number of studies show that pericytes play a key role in microvascular changes during ischemia and reperfusion. In models of cerebral, cardiac, retinal and renal injury, focal constrictions of capillaries have been observed precisely at sites where pericytes are located, suggesting that their contraction directly limits post-ischemic blood flow [114,118].

During ischemia, there is a loss of ATP and an increase in intracellular Ca^2+^, which leads to sustained contraction of pericytes; this phenomenon has also been confirmed with Ca^2+^-sensitive markers in the retina. Pharmacological blocking of Ca^2+^ channels or reduced expression of α-smooth muscle actin suppresses this contraction, confirming its active, cell-mediated nature [114,119,120].

Recent data indicate that pericyte-mediated constriction of coronary capillaries may be one of the main causes of persistently reduced flow, which has also been confirmed experimentally [121]. Additionally, it has been shown that ischemic preconditioning specifically reduces this pericyte-mediated constriction, representing one of the mechanisms of its protective role [114,122].

Interestingly, pericyte contraction, although limiting reperfusion, may also have a protective dimension - preventing excessive capillary dilation and rupture, which is associated with intramyocardial hemorrhage, the most severe manifestation of reperfusion-induced injury. This raises the question of the optimal balance between maintaining microcirculation and protecting against hemorrhage [114,123].

Pericyte-targeted therapies in preclinical models reduce capillary constriction and improve perfusion through various approaches, such as Ca^2+^ channel blockade, modulation of the endothelin and natriuretic systems, or Rho-kinase inhibition. Although clinically specific therapies are not yet available, these findings suggest that pericyte-targeted approaches could become a new strategy for preventing reperfusion injury and MVO after infarction [114].

## 10. Mechanisms of Cardioprotection

The myocardium is naturally resistant to short-term hypoxia during the normal cardiac cycle, but prolonged ischemia exceeds this protection and can cause damage. Short episodes of ischemia, known as ischemic preconditioning (IPC), represent a form of cardioprotection in which the heart is exposed to brief periods of ischemia to make the tissue more resistant to subsequent, longer periods of reduced blood flow. Initially, it was demonstrated in the heart, but today IPC can also be applied to other organs. In experimental conditions in animals, it involves occluding the blood vessel supplying the tissue for 5–10 min, after which blood flow is restored during the same period. The cycle is repeated 3–4 times before leading to prolonged ischemia that mimics an infarction [124].

At the molecular level, it has been shown that IPC acts by activating protective signaling pathways, such as the reperfusion injury salvage kinase (RISK) pathway (comprising PI3K/Akt and MAPK/ERK) and the survivor activating factor enhancement (SAFE) pathway, which involves the signal transducer and activator of transcription 3 (STAT3); these pathways, described later in the text, preserve mitochondrial function, modulate ROS production, and prevent the opening of the mPTP, thereby reducing apoptosis and necrosis [125,126]. It has also been shown to lead to a reduction in edema, leukocyte adhesion, endothelial dysfunction and MVO, while also improving vasomotion [27].

Ischemic preconditioning initially generated great interest as a method to reduce the effects of myocardial ischemia during coronary obstruction and cardiac surgeries, with intermittent aortic clamping used clinically, especially before the refinement of cardioplegic cardiac arrest [127]. Increased cardioprotection was first demonstrated in animal models through the reduction in biomarker release, while similar protective effects were observed in patients undergoing coronary artery bypass grafting with aortic clamping [128] or by applying a modified preconditioning protocol through intermittent hypoxic perfusion of the isolated heart [27].

Although IPC is not applicable in patients with acute MI, clinical and experimental studies show that similar approaches, such as ischemic postconditioning (PostC) and RIC, can reduce edema, no-reflow and intramyocardial hemorrhage and sometimes also infarct size [129].

The method that has been most studied is RIC. In studies, RIC was performed by placing a cuff on the patient’s upper arm and alternately inflating it to raise the blood pressure to 200 mmHg for 5 min, followed by deflation of the cuff for 5 min. This cycle is repeated four times, inducing brief ischemia in the arm that triggers protective mechanisms in the heart. The results of clinical RIC studies are mixed: while some studies, such as Thielmann and colleagues [130] showed improvement in biomarkers and early outcomes, two phase III prospective studies (ERICCA [131] and RIPHeart [132]) did not observe a difference in troponin release or clinical outcomes. In both studies, during cardiac surgery, patients received either the intravenous anesthetic propofol or an inhalational anesthetic. It has been shown that propofol can reduce the effects of RIC [67] while inhalational anesthetics allowed cardioprotection [133]. In STEMI patients, RIC has in many studies reduced enzymatic infarct size while some, including CONDI-2/ERIC-PPCI, did not observe a significant change [134]. White and colleagues reported a reduction in CMR-assessed infarct size, while Botker and colleagues observed an improved myocardial salvage index using single-photon emission computed tomography with RIC [27,135].

Unlike RIC, which induces cardiac protection through remote ischemic signals, PostC is applied directly to the heart immediately after the restoration of blood flow through the previously occluded artery, via short cycles of ischemia and reperfusion. In practice, this is achieved by repeatedly inflating the angioplasty balloon at the lesion site four times for one minute each, with one minute of reperfusion in between, with the possible use of contrast agent to ensure complete occlusion. In experimental models, PostC reduced infarct size, improved myocardial function and preserved the endothelium, while clinical results in STEMI patients have been mixed [136]. The combined strategy of RIC plus PostC in the LIPSIA-CONDITIONING study showed a higher myocardial salvage index and a reduction in new cases of congestive heart failure [137] while long-term analysis of CONDI-1 suggests a lower mortality rate in the RIC group [138]. Remote ischemic conditioning and PostC may be particularly beneficial in high-risk STEMI patients with hemodynamic complications, while their benefit in standard STEMI settings is limited [27,139].

Although ischemic pre-/post-conditioning and RIC show strong experimental cardioprotection, clinical effects are inconsistent. Reasons include study heterogeneity (different populations and types of interventions), variable timing (before, during, or after reperfusion), interactions with anesthesia (propofol may reduce effects, while volatile anesthetics maintain protection) and the influence of comedication (statins, beta-blockers, SGLT2i, glucagon-like peptide-1 receptor agonists). Moreover, different endpoints (infarct size, MACE) further complicate a clear interpretation of benefits. Therefore, although physiologically promising, clinical application requires precisely defined protocols and careful consideration of patient and procedural factors [27].

### 10.1. RISK and SAFE Pathways as Mechanisms of Cardioprotection

Research on cell death during the hyperacute phase of I/R injury has become a subject of investigation for identifying potential therapeutic targets. Most studies to date have focused on the classical conditioning signaling mechanisms, specifically the RISK and SAFE pathways, with the latter primarily mediated by the transcription factor STAT3 [27,140].

As previously mentioned, RIC acts through several mechanisms, including the RISK pathway as well as the SAFE and nitric oxide-dependent pathways. The involvement of these signaling cascades has been experimentally demonstrated in isolated mouse hearts [141]. Phosphoinositide 3-kinase represents a central target in cardioprotective strategies, but its function depends on the class and isoform. Class I PI3K can regulate pyroptotic mechanisms, while class III supports autophagy. To utilize PI3K for cardioprotection, it is important to emphasize that there are a large number of its isoforms. Particularly important is the α-isoform (PI3Kα), which is associated with acute cardioprotection. Gong and colleagues documented the existence of UCL-TRO-1938, a small molecule that activates PI3Kα and further aids in cardioprotection [142]. In addition to PI3K, it has been shown that overexpression of active Akt significantly delays the opening of the mPTP, indicating that activation of the PI3K-Akt prosurvival kinase pathway inhibits mPTP opening [27,125].

On the other hand, besides PI3K/Akt, activation of the extracellular signal-regulated kinase 1/2 (ERK1/2) mitogen-activated protein kinase (MAPK) signaling pathway by urocortin also protects the heart from reperfusion-induced injury in isolated rat hearts as well as in vivo. Administration of urocortin at the time of reperfusion after 35 min of ischemia reduces myocardial damage through an ERK1/ERK2 MAPK-dependent pathway. Currently available data on urocortin and ERK1/2 MAPK activation are based on preclinical models and clinical application in humans has not yet been confirmed [125].

The degree of RISK pathway activation differs among species: while the RISK pathway was necessary for activating cardioprotection and PostC in rodents and small mammals, it was not required in pigs [125].

Although activation of the RISK pathway in preclinical models has shown a significant reduction in infarct size, clinical application of these strategies has so far been disappointing. The efficacy of protection depends on the species and specific kinase isoforms (e.g., PI3Kα, Akt1 ERK1/2), while comorbidities such as diabetes or aging and the use of chronic therapy often reduce the effect, making signaling unreliable in heterogeneous patients. For example, type 2 diabetes (T2D) can abolish the cardioprotective effect of IPC by reducing Akt phosphorylation, which is further exacerbated by aging. Likewise, PostC was inadequate in rats with hypertension [125].

In addition to the RISK pathway, the alternative SAFE pathway should not be overlooked, as it also plays a key role in cardioprotection through the activation of STAT3, which acts at both the acute and subacute levels. In the subacute phase, such as late IPC or RIC 24 h before infarction, STAT3 and STAT5 increase the expression of anti-apoptotic and cytoprotective proteins (e.g., COX-2, HO-1, MnSOD, Bcl-2, c-FLIP, HSP70), supporting CMs survival and reducing tissue damage [126].

Outside the nucleus, the non-classical function of STAT3 directly contributes to acute cardiac protection by reducing infarct size and improving cardioprotective outcomes in experimental models, including mice, rats and pigs. Pharmacological blockade of STAT3 in these models shows that its activation is causally involved in infarct reduction, while basal STAT3/5 activity has no effect. Activation of the SAFE pathway and STAT3/STAT5 is associated with reduced cardiac damage and improved mitochondrial function in humans. Although mitochondrial STAT3 has not yet been directly demonstrated in the human heart, evidence from other human tissues, such as the liver and brain, suggests its existence. Therefore, it is important to further investigate its role for potential clinical applications [126].

In conclusion, the RISK and SAFE pathways are key to cardioprotection, as experimental models have shown that their activation reduces infarct size and improves mitochondrial function. In humans, activation of the RISK and SAFE pathways is associated with reduced cardiac damage and better mitochondrial function, but clinical application is limited due to patient heterogeneity, comorbidities and the lack of full confirmation of the role of mitochondrial STAT, which requires further investigation.

### 10.2. Factors That Modulate the Effectiveness of Cardioprotective Mechanisms

Patients with ischemic heart disease (IHD) often have comorbidities and classic risk factors, including T2D, hypertension, hyperlipidemia, obesity and smoking [27,46]. These factors not only accelerate the progression of coronary atherosclerosis but also increase myocardial susceptibility to I/R injury. Age-related changes in mitochondrial function contribute to left ventricular dysfunction and adverse cardiac remodeling, while increased production of ROS further reduces the effectiveness of cardioprotective interventions [46]. The following sections provide a mechanistic overview of how these individual risk factors and comorbidities interfere with cardioprotective signaling and exacerbate myocardial injury.

**Obesity and Diabetes Mellitus**

Obesity impairs cardioprotection by altering the endocrine and paracrine profile of adipose tissue. Dysfunctional adipocytes release pro-inflammatory adipokines (e.g., TNF-α, leptin, visfatin) and extracellular vesicles (EVs) that exacerbate myocardial I/R injury. Specifically, EVs from obese or diabetic models carry a distinct miRNA cargo—such as miR-130b-3p, miR-320 and miR-155—which inhibits key protective pathways (e.g., adenosine monophosphate-activated protein kinase α and PI3K/Akt/mTOR) while promoting CMs apoptosis. Conversely, EVs from lean subjects offer protection, suggesting that modulating EVs secretion or miRNA content represents a viable therapeutic strategy. Furthermore, comorbidities like T2D and aging further abolish the benefits of IPC by impairing Akt phosphorylation within the RISK pathway [125,143].

**Hypertension**

While hypertension is a prevalent risk factor, its direct impact on infarct size and I/R mortality remains controversial. However, experimental data suggest that hypertension alters exosomal miRNA profiles, specifically upregulating miR-17-5p and miR-15b-5p (which activate the pro-injury MAPK/ERK pathway) and downregulating miR-486-5p (which normally protects via PI3K/Akt/mTOR signaling). These findings indicate that hypertension increases myocardial susceptibility to injury through epigenetic shifts, though the lack of consistent human data necessitates further clinical validation [143].

**Hyperlipidemia**

Patients with hypercholesterolemia and elevated levels of oxidized LDL cholesterol have higher levels of the previously mentioned microvesicles (MVs), which enhance necrosis and fibrosis after reperfusion. The effect of hyperlipidemia on I/R injury can also be further explained by the action of statins. Statin therapy represents one of the main strategies for reducing cardiovascular risk in individuals with elevated cholesterol and its impact on I/R injury has been studied in multiple trials. Two randomized studies showed that perioperative administration of statins can reduce I/R injury, while a large study did not confirm this effect [144]. One possible mechanism of statin action is the modulation of EVs, as some authors report that statins alter their levels and composition, which may affect myocardial susceptibility to I/R injury, although this connection has not been consistently demonstrated [143,145].

Additionally, Kocsis and colleagues demonstrated in an animal model that chronic and acute therapy with lovastatin have different effects on conditioning mechanisms: chronic therapy reduces the efficacy of preconditioning, while acute therapy reduces the efficacy of PostC. In patients treated with statins (simvastatin, rosuvastatin, atorvastatin), a lower number of MVs, particularly those derived from platelets, leukocytes and endothelial cells, was observed compared to untreated patients [145]. This effect was not observed in diabetic patients receiving pitavastatin therapy [143].

Moreover, the effect of statins on MVs release is time- and dose-dependent: it has been shown that 40 mg of atorvastatin reduces the number of MVs significantly more than 10 mg after one year of treatment [145]. These data suggest that MVs may play an important role in I/R injury, but their function depends on their origin and content, not just their number [143] (Figure 3).

**Chronic Kidney Disease**

Chronic kidney disease (CKD) can significantly reduce the heart’s ability to protect itself from injury, primarily through the accumulation of uremic toxins and metabolic disturbances that disrupt normal cardiac and vascular functions. Uremic toxins (e.g., indoxyl sulfate, p-cresol sulfates) promote oxidative stress, NOX activation and reduced antioxidant capacity, leading to endothelial dysfunction, cardiac myocyte injury and an increased risk of hypertrophy, fibrosis and cardiac dysfunction [146]. Additionally, in CKD, chronic inflammation, mineral metabolism disturbances (Ca^2+^, phosphate), anemia and fluid retention are common, further burdening the heart [147].

**Chronic Inflammation**

Chronic inflammation reduces the heart’s ability to activate protective adaptive mechanisms, as prolonged elevated activity of immune and inflammatory pathways in the cardiovascular system disrupts the balance between protective and damaging processes. Elevated levels of pro-inflammatory cytokines, such as TNF-α, IL-6 and IL-1β, contribute to oxidative stress, endothelial cell damage and cardiac remodeling, including hypertrophy and fibrosis [148]. Chronic inflammation further weakens the heart’s protective mechanisms by disrupting cardioprotective signaling pathways, including PI3K/Akt and reperfusion preadaptation, reducing CMs mitochondrial function and enhancing activation of pro-apoptotic pathways, making the heart more susceptible to I/R injury and decreasing its regenerative potential [149].

**Smoking**

Smoking accelerates MI onset by approximately a decade and is strongly associated with intramyocardial hemorrhage and microvascular damage [150]. Acute exposure triggers an immediate increase in MVs production, likely driven by smoke-induced cellular Ca^2+^ elevation [151]. These EVs often carry pro-inflammatory cytokines (IL-1, IL-6, IL-8), matrix metalloproteinase-14 and tissue factor, which exacerbate I/R injury by activating the inflammasome and pyroptosis [152]. Furthermore, smoke modulates EV-miRNA profiles: miR-210 is upregulated, worsening I/R injury via autophagy inhibition [153], while miR-126 and miR-125a may offer protection by reducing apoptosis [154]. The role of other molecules, such as miR-223, remains controversial due to contradictory effects on oxidative stress and necroptosis. Given the inconsistent data across studies, a context-dependent interpretation of miRNA roles in smokers is essential to avoid overgeneralization [155].

**Aging**

Aging alters the composition and function of EVs, increasing myocardial susceptibility to I/R injury. In older individuals, EVs are enriched in pro-inflammatory molecules and miRNAs such as miR-128-3p, while levels of cardioprotective components, including mtDNA and galectin-3, decrease, leading to increased oxidative stress, apoptosis and fibrosis. These changes reduce the effectiveness of cardioprotective pathways, including RISK and antioxidant mechanisms, contributing to greater cardiac vulnerability in the elderly [143].

**Sex**

Sexual dimorphism in mitochondrial functions (mPTP, oxidative stress and respiratory activities) can influence the response to I/R injury; for example, women with STEMI show greater myocardial preservation compared to men. Similarly, in premenopausal rodents, the female sex exerts a protective effect due to estrogen, whereas in pigs these differences are not significant [27].

**Use of Medications**

Patients with IHD are often older and have multiple comorbidities requiring chronic therapy (antidiabetics, antihypertensives, lipid-lowering drugs, P2Y_12_ inhibitors, opioids and anesthetics). Drugs such as statins and their cardioprotective effects have been described above. Other medications, including nitroglycerin and morphine, have also demonstrated protective effects, whereas some, such as sulfonylureas, interfere with cardioprotective signaling pathways [46]. In our review, the inhibitory effect of propofol on remote PostC was also mentioned [27,67]. Additionally, the use of P2Y_12_ inhibitors ticagrelor and prasugrel in STEMI patients after PCI is associated with a reduction in major cardiovascular events, cardiovascular mortality and all-cause mortality, indicating their clinically relevant cardioprotective effect [156] (Figure 3).

## 11. Current Translational Directions in the Therapy of Ischemia-Reperfusion Injury

Current translational approaches in the therapy of I/R injury are focused on regulating key metabolic and inflammatory processes that determine CMs fate during reperfusion.

One of the most extensively studied strategies is targeting succinate and RET at Complex I, as rapid succinate oxidation upon oxygen reflow triggers a strong production of ROS [27]. In this context, malonate has been shown to be an effective SDH inhibitor, reducing succinate accumulation during ischemia [157] while MitoSNO, through short-term S-nitrosylation of Complex I, prevents RET-mediated oxidative damage during reperfusion. Additionally, mitochondria-targeted antioxidants, such as MitoQ and plastoquinonyl-decyl-triphenylphosphonium, have been developed [158] which selectively accumulate in mitochondria and efficiently neutralize ROS at their site of production.

Maintaining ionic homeostasis represents another key target in translational strategies. Inhibition of the NCX (e.g., cariporide) reduces Na^+^ accumulation during ischemia, thereby indirectly preventing Ca^2+^ overload [33]. Similarly, modulation of the NCX plays an important role, especially during reperfusion when NCX can bring Ca^2+^ into the cell in reverse mode. SEA0400 is a classical pharmacological NCX inhibitor, while newer compounds such as ORM-10962 demonstrate greater selectivity and efficacy, as they more precisely block the reverse mode of NCX without significantly affecting other ion currents, making them promising candidates for preventing Ca^2+^ overload [27,35].

Further translational directions include CaMKII inhibitors, such as KN-93, since activation of this kinase by ROS contributes to necroptosis, arrhythmias and mPTP opening [159]. Complement-targeted therapies, including C1 inhibition [160], or C5 (e.g., eculizumab) [161], help control the early inflammatory cascade. Finally, antithromboinflammatory strategies, such as P2Y_12_ inhibitors (ticagrelor), mitigate the reciprocal amplification of thrombotic and inflammatory responses, further reducing myocardial damage [156].

### 11.1. Antioxidants and Xanthine Oxidase Inhibitors

Although preclinical data are promising, the efficacy of antioxidants and XO inhibitors in preventing I/R injury faces numerous limitations, which will be discussed below.

Primarily, oxidative stress is a highly dynamic process that peaks during the reperfusion phase—a period known as the “oxidative burst.” Untimely administration of these drugs, either before or after this critical moment, significantly reduces their ability to neutralize ROS [20].

Secondly, systemically administered antioxidants and XO inhibitors often fail to reach adequate concentrations at the site of myocardial injury, primarily due to poor pharmacokinetics, rapid elimination and lack of targeted delivery to affected cells, which limits their effectiveness. Moreover, XO is only one of many sources of ROS in injured tissue, so inhibition of a single enzyme is often insufficient to significantly reduce oxidative stress [20].

Thirdly, drugs may not reach the precise subcellular locations where ROS are generated, so XO inhibition can reduce ROS originating from purine metabolism while other sources (mitochondria, NOX) continue to produce ROS and antioxidants do not reach these sites (e.g., mitochondria), further diminishing the therapeutic effect [20].

What further complicates achieving a unified therapeutic goal is patient heterogeneity in terms of age, comorbidities and genetic predisposition [162].

These factors, combined with individual patient differences and the complexity of redox processes, explain why antioxidants and XO inhibitors have not achieved the expected clinical success despite promising preclinical results. It should also not be overlooked that, besides causing oxidative stress and cellular damage, ROS are important signaling molecules. Therefore, nonselective and excessive removal of ROS can disrupt this balance and lead to harmful consequences [20].

### 11.2. Translation of Preclinical Mitochondrial and Pharmacological Strategies into Clinical Practice

Preclinical studies have shown that various drugs and molecular strategies targeting mitochondria can potentially reduce infarct size and improve mitochondrial function in I/R injury models. Among these approaches, notable ones include SDH inhibition (e.g., with malonate), regulation of mitochondrial dynamics through fission inhibition (e.g., mitochondrial division inhibitor-1 [Mdivi-1], Drpitor1a, and peptide P110), and the use of mitochondrial peptides such as Szeto-Schiller peptides, elamipretide (MTP-131) and mitochondria-derived peptide humanin analogue HNG. These compounds have demonstrated in experimental models a reduction in oxidative stress, mitochondrial protection, decreased apoptosis and necrosis, as well as limitation of infarct size [46].

However, when it comes to clinical applications, the results are limited. For example, the use of elamipretide in patients with a first anterior STEMI undergoing PCI did not significantly reduce infarct size. Similarly, a pilot study with Mdivi-1 in a porcine acute MI model did not lead to a measurable reduction in infarct size or improvement in cardiac function, which can be attributed to insufficient dose or the method of drug delivery [163]. Thus, despite promising preclinical results, the translation of these strategies to the clinical level has still not resulted in effective therapy for reducing infarct size, highlighting the need for further research and optimization of these approaches [46].

The timely administration of certain cardioprotective drugs during the acute ischemic phase demonstrates clear translational value. Intravenous metoprolol, in particular, stands out in STEMI patients undergoing primary PCI, where it has been associated with reduced infarct size, decreased incidence of ventricular fibrillation and microvascular complications and improved systolic function [46]. Experimental studies with propranolol in dogs [164] and with landiolol in pigs indicate that β-blockade acts by reducing Ca^2+^ influx and inhibiting contractile-induced myocardial injury [165] which supports the translation of experimental models to clinical practice [46].

These findings indicate that the translation of preclinical strategies into clinical practice is successful for β-blockers, whereas for other therapies, such as Ca^2+^ channel blockers or selective myosin inhibitors (mavacamten, aficamten), there remains a significant gap between laboratory and clinical outcomes [46,166].

## 12. Conclusions

Myocardial I/R injury represents a complex pathophysiological process that activates a wide range of interconnected molecular mechanisms, including various forms of cell death, oxidative stress, mitochondrial dysfunction, inflammatory responses and metabolic disturbances. These mechanisms act simultaneously and synergistically, with each contributing in a specific manner to CM injury and cardiac remodeling, which significantly complicates the development of optimal therapeutic strategies. Understanding the temporal dynamics and predominant influence of individual mechanisms is crucial for precisely targeting therapeutic interventions toward specific signaling pathways, thereby achieving greater efficacy in the prevention and mitigation of I/R injury. Despite significant progress in elucidating the underlying pathobiological processes, further research is needed to develop innovative and reliable cardioprotective therapies that can reduce the long-term complications and mortality resulting from myocardial I/R injury.

## Data Availability

No new data were created or analyzed in this study.

## References

[1] Schirone L., Forte M., D’ambrosio L., Valenti V., Vecchio D., Schiavon S., Spinosa G., Sarto G., Petrozza V., Frati G. (2022). An Overview of the Molecular Mechanisms Associated with Myocardial Ischemic Injury: State of the Art and Translational Perspectives. Cells.

[2] Pepine C.J., Nichols W.W. (2007). The pathophysiology of chronic ischemic heart disease. Clin. Cardiol..

[3] Buja L.M., Vander Heide R.S. (2016). Pathobiology of Ischemic Heart Disease: Past, Present and Future. Cardiovasc. Pathol..

[4] Buja L.M. (2023). Pathobiology of Myocardial Ischemia and Reperfusion Injury: Models, Modes, Molecular Mechanisms, Modulation, and Clinical Applications. Cardiol. Rev..

[5] Hausenloy D.J., Yellon D.M. (2013). Myocardial ischemia-reperfusion injury: A neglected therapeutic target. J. Clin. Investig..

[6] Jennings R.B. (2013). Historical Perspective on the Pathology of Myocardial Ischemia/Reperfusion Injury. Circ. Res..

[7] Jennings R.B., Reimer K.A. (1991). The Cell Biology of Acute Myocardial Ischemia. Annu. Rev. Med..

[8] Liu Y., Li L., Wang Z., Zhang J., Zhou Z. (2023). Myocardial ischemia-reperfusion injury; Molecular mechanisms and prevention. Microvasc. Res..

[9] Davidson S.M., Adameová A., Barile L., Cabrera-Fuentes H.A., Lazou A., Pagliaro P., Stensløkken K., Garcia-Dorado D., Action E.-C.C. (2020). Mitochondrial and mitochondrial-independent pathways of myocardial cell death during ischaemia and reperfusion injury. J. Cell. Mol. Med..

[10] Maximilian Buja L., Vela D. (2008). Cardiomyocyte death and renewal in the normal and diseased heart. Cardiovasc. Pathol..

[11] Weerasinghe P., Buja L.M. (2012). Oncosis: An important non-apoptotic mode of cell death. Exp. Mol. Pathol..

[12] Bertero E., Popoiu T.-A., Maack C. (2024). Mitochondrial calcium in cardiac ischemia/reperfusion injury and cardioprotection. Basic Res. Cardiol..

[13] Fan M., Zhang J., Tsai C.-W., Orlando B.J., Rodriguez M., Xu Y., Liao M., Tsai M.-F., Feng L. (2020). Structure and mechanism of the mitochondrial Ca2+ uniporter holocomplex. Nature.

[14] Maack C., Cortassa S., Aon M.A., Ganesan A.N., Liu T., O’Rourke B. (2006). Elevated Cytosolic Na ^+^ Decreases Mitochondrial Ca^2+^ Uptake During Excitation-Contraction Coupling and Impairs Energetic Adaptation in Cardiac Myocytes. Circ. Res..

[15] Hartmann M., Decking U.K.M. (1999). Blocking Na^+^–H^+^Exchange by Cariporide Reduces Na^+^-overload in Ischemia and is Cardioprotective. J. Mol. Cell. Cardiol..

[16] Boyman L., Coleman A.K., Zhao G., Wescott A.P., Joca H.C., Greiser B.M., Karbowski M., Ward C.W., Lederer W. (2019). Dynamics of the mitochondrial permeability transition pore: Transient and permanent opening events. Arch. Biochem. Biophys..

[17] Endlicher R., Drahota Z., Štefková K., Červinková Z., Kučera O. (2023). The Mitochondrial Permeability Transition Pore—Current Knowledge of Its Structure, Function, and Regulation, and Optimized Methods for Evaluating Its Functional State. Cells.

[18] Hausenloy D.J., Yellon D.M. (2016). Ischaemic conditioning and reperfusion injury. Nat. Rev. Cardiol..

[19] Thomas K.S., Puthooran D.M., Edpuganti S., Reddem A.L., Jose A., Akula S.S.M. (2025). Reperfusion injury in STEMI: A double-edged sword. Egypt. Heart J..

[20] Chen H., Tang Y., Ren P., Wu W. (2025). The unmet promise: A critical review of antioxidant strategies in myocardial ischemia-reperfusion injury and the path towards precision medicine. Front. Pharmacol..

[21] Duilio C., Ambrosio G., Kuppusamy P., DiPaula A., Becker L.C., Zweier J.L. (2001). Neutrophils are primary source of O_2_ radicals during reperfusion after prolonged myocardial ischemia. Am. J. Physiol. Heart Circ. Physiol..

[22] Koshinuma S., Miyamae M., Kaneda K., Kotani J., Figueredo V.M. (2014). Combination of necroptosis and apoptosis inhibition enhances cardioprotection against myocardial ischemia-reperfusion injury. J. Anesth..

[23] Reffelmann T., Kloner R.A. (2002). Microvascular reperfusion injury: Rapid expansion of anatomic no reflow during reperfusion in the rabbit. Am. J. Physiol. Heart Circ. Physiol..

[24] Hausenloy D. (2003). Inhibiting mitochondrial permeability transition pore opening at reperfusion protects against ischaemia–reperfusion injury. Cardiovasc. Res..

[25] Asimakis G.K., Lick S., Patterson C. (2002). Postischemic Recovery of Contractile Function is Impaired in SOD2^+/−^ but Not SOD1^+/−^ Mouse Hearts. Circulation.

[26] Zaha V.G., Qi D., Su K.N., Palmeri M., Lee H.-Y., Hu X., Wu X., Shulman G.I., Rabinovitch P.S., Russell R.R. (2016). AMPK is critical for mitochondrial function during reperfusion after myocardial ischemia. J. Mol. Cell. Cardiol..

[27] Heusch G., Andreadou I., Bell R., Bertero E., Botker H.-E., Davidson S.M., Downey J., Eaton P., Ferdinandy P., Gersh B.J. (2023). Health position paper and redox perspectives on reactive oxygen species as signals and targets of cardioprotection. Redox Biol..

[28] Penzo D., Petronilli V., Angelin A., Cusan C., Colonna R., Scorrano L., Pagano F., Prato M., Di Lisa F., Bernardi P. (2004). Arachidonic Acid Released by Phospholipase A2 Activation Triggers Ca^2+^-dependent Apoptosis through the Mitochondrial Pathway. J. Biol. Chem..

[29] Daiber A., Di Lisa F., Oelze M., Kröller-Schön S., Steven S., Schulz E., Münzel T. (2017). Crosstalk of mitochondria with NADPH oxidase via reactive oxygen and nitrogen species signalling and its role for vascular function. Br. J. Pharmacol..

[30] Liu G.-Y., Xie W.-L., Wang Y.-T., Chen L., Xu Z.-Z., Lv Y., Wu Q.-P. (2023). Calpain: The regulatory point of myocardial ischemia-reperfusion injury. Front. Cardiovasc. Med..

[31] Robb E.L., Gawel J.M., Aksentijević D., Cochemé H.M., Stewart T.S., Shchepinova M.M., Qiang H., Prime T.A., Bright T.P., James A.M. (2015). Selective superoxide generation within mitochondria by the targeted redox cycler MitoParaquat. Free Radic. Biol. Med..

[32] Wojtovich A.P., Smith C.O., Haynes C.M., Nehrke K.W., Brookes P.S. (2013). Physiological consequences of complex II inhibition for aging, disease, and the mKATP channel. Biochim. Biophys. Acta (BBA) Bioenerg..

[33] Zhang Y., Chen J., Zhang F., Xia Q. (2006). Cariporide attenuates myocardial ischaemia and reperfusion injury and apoptosis in isolated rat hearts. Acta Cardiol..

[34] Chen W., Zhang Y., Wang Z., Tan M., Lin J., Qian X., Li H., Jiang T. (2023). Dapagliflozin alleviates myocardial ischemia/reperfusion injury by reducing ferroptosis via MAPK signaling inhibition. Front. Pharmacol..

[35] Ismaili D., Gurr K., Horváth A., Yuan L., Lemoine M.D., Schulz C., Sani J., Petersen J., Reichenspurner H., Kirchhof P. (2022). Regulation of APD and Force by the Na^+^/Ca^2+^ Exchanger in Human-Induced Pluripotent Stem Cell-Derived Engineered Heart Tissue. Cells.

[36] de J. García-Rivas G., Carvajal K., Correa F., Zazueta C. (2006). Ru_360_, a specific mitochondrial calcium uptake inhibitor, improves cardiac post-ischaemic functional recovery in rats in vivo. Br. J. Pharmacol..

[37] Yingzhong C., Lin C., Chunbin W. (2016). Clinical effects of cyclosporine A on reperfusion injury in myocardial infarction: A meta-analysis of randomized controlled trials. SpringerPlus.

[38] Chouchani E.T., Pell V.R., Gaude E., Aksentijević D., Sundier S.Y., Robb E.L., Logan A., Nadtochiy S.M., Ord E.N.J., Smith A.C. (2014). Ischaemic accumulation of succinate controls reperfusion injury through mitochondrial ROS. Nature.

[39] Mills E.L., Pierce K.A., Jedrychowski M.P., Garrity R., Winther S., Vidoni S., Yoneshiro T., Spinelli J.B., Lu G.Z., Kazak L. (2018). Accumulation of succinate controls activation of adipose tissue thermogenesis. Nature.

[40] Zhang J., Wang Y.T., Miller J.H., Day M.M., Munger J.C., Brookes P.S. (2018). Accumulation of Succinate in Cardiac Ischemia Primarily Occurs via Canonical Krebs Cycle Activity. Cell Rep..

[41] Chen Q., Moghaddas S., Hoppel C.L., Lesnefsky E.J. (2006). Reversible Blockade of Electron Transport during Ischemia Protects Mitochondria and Decreases Myocardial Injury following Reperfusion. J. Pharmacol. Exp. Ther..

[42] Kula-Alwar D., Prag H.A., Krieg T. (2019). Targeting Succinate Metabolism in Ischemia/Reperfusion Injury. Circulation.

[43] Kwong J.Q., Molkentin J.D. (2015). Physiological and Pathological Roles of the Mitochondrial Permeability Transition Pore in the Heart. Cell Metab..

[44] Zhang C.-X., Cheng Y., Liu D.-Z., Liu M., Cui H., Zhang B.-L., Mei Q.-B., Zhou S.-Y. (2019). Mitochondria-targeted cyclosporin A delivery system to treat myocardial ischemia reperfusion injury of rats. J. Nanobiotechnol..

[45] Malouitre S., Dube H., Selwood D., Crompton M. (2009). Mitochondrial targeting of cyclosporin A enables selective inhibition of cyclophilin-D and enhanced cytoprotection after glucose and oxygen deprivation. Biochem. J..

[46] Paillard M., Abdellatif M., Andreadou I., Bär C., Bertrand L., Brundel B.J., Chiva-Blanch G., Davidson S.M., Dawson D., Di Lisa F. (2025). Mitochondrial targets in ischaemic heart disease and heart failure, and their potential for a more efficient clinical translation. A scientific statement of the ESC Working Group on Cellular Biology of the Heart and the ESC Working Group on Myocardial Function. Eur. J. Heart Fail..

[47] Hernando V., Inserte J., Sartório C.L., Parra V.M., Poncelas-Nozal M., Garcia-Dorado D. (2010). Calpain translocation and activation as pharmacological targets during myocardial ischemia/reperfusion. J. Mol. Cell. Cardiol..

[48] Williams S.D., Gottlieb R.A. (2002). Inhibition of mitochondrial calcium-independent phospholipase A2 (iPLA2) attenuates mitochondrial phospholipid loss and is cardioprotective. Biochem. J..

[49] Badimon L., Vilahur G. (2014). Thrombosis formation on atherosclerotic lesions and plaque rupture. J. Intern. Med..

[50] Gupta S., Knowlton A.A. (2007). HSP60 trafficking in adult cardiac myocytes: Role of the exosomal pathway. Am. J. Physiol. Heart Circ. Physiol..

[51] Lin L., Knowlton A.A. (2014). Innate immunity and cardiomyocytes in ischemic heart disease. Life Sci..

[52] Tian J., Guo X., Liu X.-M., Liu L., Weng Q.-F., Dong S.-J., Knowlton A.A., Yuan W.-J., Lin L. (2013). Extracellular HSP60 induces inflammation through activating and up-regulating TLRs in cardiomyocytes. Cardiovasc. Res..

[53] Xu H., Yao Y., Su Z., Yang Y., Kao R., Martin C.M., Rui T. (2011). Endogenous HMGB1 contributes to ischemia-reperfusion-induced myocardial apoptosis by potentiating the effect of TNF-α/JNK. Am. J. Physiol. Heart Circ. Physiol..

[54] Juricic S., Klac J., Stojkovic S., Tesic M., Jovanovic I., Aleksandric S., Dobric M., Zivkovic S., Maricic B., Simeunovic D. (2025). Molecular and Pathophysiological Mechanisms Leading to Ischemic Heart Disease in Patients with Diabetes Mellitus. Int. J. Mol. Sci..

[55] Linfert D., Chowdhry T., Rabb H. (2009). Lymphocytes and ischemia-reperfusion injury. Transplant. Rev..

[56] Nimmerjahn F., Ravetch J.V. (2010). Antibody-mediated modulation of immune responses. Immunol. Rev..

[57] Hofmann U., Frantz S. (2015). Role of lymphocytes in myocardial injury, healing, and remodeling after myocardial infarction. Circ. Res..

[58] Wang Y.-P., Xie Y., Ma H., Su S.-A., Wang Y.-D., Wang J.-A., Xiang M.-X. (2016). Regulatory T lymphocytes in myocardial infarction: A promising new therapeutic target. Int. J. Cardiol..

[59] van der Laan A.M., Ter Horst E.N., Delewi R., Begieneman M.P., Krijnen P.A., Hirsch A., Lavaei M., Nahrendorf M., Horrevoets A.J., Niessen H.W. (2014). Monocyte subset accumulation in the human heart following acute myocardial infarction and the role of the spleen as monocyte reservoir. Eur. Heart J..

[60] Heusch G. (2024). Myocardial ischemia/reperfusion: Translational pathophysiology of ischemic heart disease. Med.

[61] Heusch G., Kleinbongard P. (2025). The spleen in ischaemic heart disease. Nat. Rev. Cardiol..

[62] Carnevale D. (2022). Neuroimmune axis of cardiovascular control: Mechanisms and therapeutic implications. Nat. Rev. Cardiol..

[63] Lieder H.R., Paket U., Skyschally A., Rink A.D., Baars T., Neuhäuser M., Kleinbongard P., Heusch G. (2024). Vago-splenic signal transduction of cardioprotection in humans. Eur. Heart J..

[64] Lieder H.R., Kleinbongard P., Skyschally A., Hagelschuer H., Chilian W.M., Heusch G. (2018). Vago-Splenic Axis in Signal Transduction of Remote Ischemic Preconditioning in Pigs and Rats. Circ. Res..

[65] Gao X.-M., Moore X.-L., Liu Y., Wang X.-Y., Han L.-P., Su Y., Tsai A., Xu Q., Zhang M., Lambert G.W. (2016). Splenic release of platelets contributes to increased circulating platelet size and inflammation after myocardial infarction. Clin. Sci..

[66] Huang Z., Qian C., Zhang Z., Nian W., Xu Q., Cao Y., Fu C. (2024). Ticagrelor regulates the differentiation of MDSCs after acute myocardial infarction to reduce cardiac injury. Biomed. Pharmacother..

[67] Kottenberg E., Thielmann M., Bergmann L., Heine T., Jakob H., Heusch G., Peters J. (2012). Protection by remote ischemic preconditioning during coronary artery bypass graft surgery with isoflurane but not propofol—A clinical trial. Acta Anaesthesiol. Scand..

[68] Piper H. (1998). A fresh look at reperfusion injury. Cardiovasc. Res..

[69] Piper H.M., Abdallah Y., Schäfer C. (2004). The first minutes of reperfusion: A window of opportunity for cardioprotection. Cardiovasc. Res..

[70] Rodríguez-Sinovas A., Abdallah Y., Piper H.M., Garcia-Dorado D. (2007). Reperfusion injury as a therapeutic challenge in patients with acute myocardial infarction. Heart Fail. Rev..

[71] Yusof N.L.M., Yellon D.M., Davidson S.M. (2025). The contribution of cardiomyocyte hypercontracture to the burden of acute myocardial infarction: An update. Basic Res. Cardiol..

[72] Ladilov Y. (2003). Reoxygenation-induced rigor-type contracture. J. Mol. Cell. Cardiol..

[73] Inserte J., Garcia-Dorado D., Ruiz-Meana M., Padilla F., Barrabés J.A., Pina P., Agulló L., Piper H.M., Soler-Soler J. (2002). Effect of inhibition of Na^+^/Ca^2+^ exchanger at the time of myocardial reperfusion on hypercontracture and cell death. Cardiovasc. Res..

[74] Maron M.S., Masri A., Choudhury L., Olivotto I., Saberi S., Wang A., Garcia-Pavia P., Lakdawala N.K., Nagueh S.F., Rader F. (2023). Phase 2 Study of Aficamten in Patients With Obstructive Hypertrophic Cardiomyopathy. J. Am. Coll. Cardiol..

[75] Tsurusaki S., Kizana E. (2024). Mechanisms and Therapeutic Potential of Multiple Forms of Cell Death in Myocardial Ischemia-Reperfusion Injury. Int. J. Mol. Sci..

[76] Fischer U.M., Tossios P., Huebner A., Geissler H.J., Bloch W., Mehlhorn U. (2004). Myocardial apoptosis prevention by radical scavenging in patients undergoing cardiac surgery. J. Thorac. Cardiovasc. Surg..

[77] Wang K., Zhu Q., Liu W., Wang L., Li X., Zhao C., Wu N., Ma C. (2025). Mitochondrial apoptosis in response to cardiac ischemia-reperfusion injury. J. Transl. Med..

[78] Freude B., Masters T.N., Robicsek F., Fokin A., Kostin S., Zimmermann R., Ullmann C., Lorenz-Meyer S., Schaper J. (2000). Apoptosis is Initiated by Myocardial Ischemia and Executed During Reperfusion. J. Mol. Cell. Cardiol..

[79] Wang J., Toan S., Zhou H. (2020). Mitochondrial quality control in cardiac microvascular ischemia-reperfusion injury: New insights into the mechanisms and therapeutic potentials. Pharmacol. Res..

[80] Vila-Julià G., Perez J.J., Rubio-Martinez J. (2023). A Step Forward toward Selective Activation/Inhibition of Bak, a Pro-Apoptotic Member of the Bcl-2 Protein Family: Discovery of New Prospective Allosteric Sites Using Molecular Dynamics. J. Chem. Inf. Model..

[81] Huang W., Yang J., He C., Yang J. (2020). RP105 plays a cardioprotective role in myocardial ischemia reperfusion injury by regulating the Toll-like receptor 2/4 signaling pathways. Mol. Med. Rep..

[82] Liu M., Lv J., Pan Z., Wang D., Zhao L., Guo X. (2022). Mitochondrial dysfunction in heart failure and its therapeutic implications. Front. Cardiovasc. Med..

[83] Wu C., Zhang Z., Zhang W., Liu X. (2022). Mitochondrial dysfunction and mitochondrial therapies in heart failure. Pharmacol. Res..

[84] Hu Y., Pan H., Peng J., He J., Tang M., Yan S., Rong J., Li J., Zheng Z., Wang H. (2021). Resveratrol inhibits necroptosis by mediating the TNF-α/RIP1/RIP3/MLKL pathway in myocardial hypoxia/reoxygenation injury. Acta Biochim. Biophysc. Sin..

[85] Cao L., Mu W. (2021). Necrostatin-1 and necroptosis inhibition: Pathophysiology and therapeutic implications. Pharmacol. Res..

[86] Takahashi N., Duprez L., Grootjans S., Cauwels A., Nerinckx W., DuHadaway J.B., Goossens V., Roelandt R., Van Hauwermeiren F., Libert C. (2012). Necrostatin-1 analogues: Critical issues on the specificity, activity and in vivo use in experimental disease models. Cell Death Dis..

[87] Zeng W., Cao Y., Jiang W., Kang G., Huang J., Xie S. (2019). Knockdown of Sfrp4 attenuates apoptosis to protect against myocardial ischemia/reperfusion injury. J. Pharmacol. Sci..

[88] Zhu H., Tan Y., Du W., Li Y., Toan S., Mui D., Tian F., Zhou H. (2021). Phosphoglycerate mutase 5 exacerbates cardiac ischemia-reperfusion injury through disrupting mitochondrial quality control. Redox Biol..

[89] She L., Tu H., Zhang Y.-Z., Tang L.-J., Li N.-S., Ma Q.-L., Liu B., Li Q., Luo X.-J., Peng J. (2019). Inhibition of Phosphoglycerate Mutase 5 Reduces Necroptosis in Rat Hearts Following Ischemia/Reperfusion Through Suppression of Dynamin-Related Protein 1. Cardiovasc. Drugs Ther..

[90] Lu W., Sun J., Yoon J.S., Zhang Y., Zheng L., Murphy E., Mattson M.P., Lenardo M.J. (2016). Mitochondrial Protein PGAM5 Regulates Mitophagic Protection against Cell Necroptosis. PLoS ONE.

[91] Zhang L., Hu Z., Li Z., Lin Y. (2024). Crosstalk among mitophagy, pyroptosis, ferroptosis, and necroptosis in central nervous system injuries. Neural Regen. Res..

[92] Zhou G., Wu H., Yang J., Ye M., Liu D., Li Y., Zhang D., Zhang J., Yang Q., Liu Y. (2023). Liraglutide Attenuates Myocardial Ischemia/Reperfusion Injury Through the Inhibition of Necroptosis by Activating GLP-1R/PI3K/Akt Pathway. Cardiovasc. Toxicol..

[93] Zhu P., Hu S., Jin Q., Li D., Tian F., Toan S., Li Y., Zhou H., Chen Y. (2018). Ripk3 promotes ER stress-induced necroptosis in cardiac IR injury: A mechanism involving calcium overload/XO/ROS/mPTP pathway. Redox Biol..

[94] Li J., Zhang J., Zhong Y., Xie D., Han H., Zhang Z., Liu Y., Li S. (2024). TRPC6 regulates necroptosis in myocardial ischemia/reperfusion injury via Ca2+/CaMKII signaling pathway. Cell. Signal..

[95] Mishra P.K., Adameova A., Hill J.A., Baines C.P., Kang P.M., Downey J.M., Narula J., Takahashi M., Abbate A., Piristine H.C. (2019). Guidelines for evaluating myocardial cell death. Am. J. Physiol. Heart Circ. Physiol..

[96] Broz P. (2025). Pyroptosis: Molecular mechanisms and roles in disease. Cell Res..

[97] He W.-T., Wan H., Hu L., Chen P., Wang X., Huang Z., Yang Z.-H., Zhong C.-Q., Han J. (2015). Gasdermin D is an executor of pyroptosis and required for interleukin-1β secretion. Cell Res..

[98] Merkle S., Frantz S., Schön M.P., Bauersachs J., Buitrago M., Frost R.J., Schmitteckert E.M., Lohse M.J., Engelhardt S. (2007). A Role for Caspase-1 in Heart Failure. Circ. Res..

[99] Wu X., Hou Y.-L., Wang T.-X., Chang L.-P., Zhou H.-R., Wang M.-Y., Wu Y.-L. (2025). Tongxinluo alleviates myocardial ischemia–reperfusion injury by inhibiting the pyroptosis of endothelial cells via the NLRP3/Caspase-1/GSDMD signaling pathway. J. Mol. Histol..

[100] Figueiredo-Pereira C., Dias-Pedroso D., Soares N.L., Vieira H.L.A. (2020). CO-mediated cytoprotection is dependent on cell metabolism modulation. Redox Biol..

[101] Seiler D.K., Hay J.C. (2022). Genetically encoded fluorescent tools: Shining a little light on ER-to-Golgi transport. Free Radic. Biol. Med..

[102] Dabkowski E.R., Williamson C.L., Hollander J.M. (2008). Mitochondria-specific transgenic overexpression of phospholipid hydroperoxide glutathione peroxidase (GPx4) attenuates ischemia/reperfusion-associated cardiac dysfunction. Free Radic. Biol. Med..

[103] Pan Y., Wang X., Liu X., Shen L., Chen Q., Shu Q. (2022). Targeting Ferroptosis as a Promising Therapeutic Strategy for Ischemia-Reperfusion Injury. Antioxidants.

[104] Feng Y., Madungwe N.B., Imam Aliagan A.D., Tombo N., Bopassa J.C. (2019). Liproxstatin-1 protects the mouse myocardium against ischemia/reperfusion injury by decreasing VDAC1 levels and restoring GPX4 levels. Biochem. Biophys. Res. Commun..

[105] Aljakna Khan A., Sabatasso S. (2025). Autophagy in myocardial ischemia and ischemia/reperfusion. Cardiovasc. Pathol..

[106] González A., Hall M.N., Lin S.-C., Hardie D.G. (2020). AMPK and TOR: The Yin and Yang of Cellular Nutrient Sensing and Growth Control. Cell Metab..

[107] Deng J., Liu Q., Ye L., Wang S., Song Z., Zhu M., Qiang F., Zhou Y., Guo Z., Zhang W. (2024). The Janus face of mitophagy in myocardial ischemia/reperfusion injury and recovery. Biomed. Pharmacother..

[108] Hollville E., Carroll R.G., Cullen S.P., Martin S.J. (2014). Bcl-2 family proteins participate in mitochondrial quality control by regulating Parkin/PINK1-dependent mitophagy. Mol. Cell.

[109] Bingol B., Tea J.S., Phu L., Reichelt M., Bakalarski C.E., Song Q., Foreman O., Kirkpatrick D.S., Sheng M. (2014). The mitochondrial deubiquitinase USP30 opposes parkin-mediated mitophagy. Nature.

[110] Panisello-Roselló A., Alva N., Flores M., Lopez A., Benítez C.C., Folch-Puy E., Rolo A., Palmeira C., Adam R., Carbonell T. (2018). Aldehyde Dehydrogenase 2 (ALDH2) in Rat Fatty Liver Cold Ischemia Injury. Int. J. Mol. Sci..

[111] Wang Z., Lin D., Cui B., Zhang D., Wu J., Ma J. (2024). Melatonin protects against myocardial ischemia-reperfusion injury by inhibiting excessive mitophagy through the Apelin/SIRT3 signaling axis. Eur. J. Pharmacol..

[112] Liu X.-W., Lu M.-K., Zhong H.-T., Wang L.-H., Fu Y.-P. (2019). Panax Notoginseng Saponins Attenuate Myocardial Ischemia-Reperfusion Injury Through the HIF-1α/BNIP3 Pathway of Autophagy. J. Cardiovasc. Pharmacol..

[113] de Waha S., Patel M.R., Granger C.B., Ohman E.M., Maehara A., Eitel I., Ben-Yehuda O., Jenkins P., Thiele H., Stone G.W. (2017). Relationship between microvascular obstruction and adverse events following primary percutaneous coronary intervention for ST-segment elevation myocardial infarction: An individual patient data pooled analysis from seven randomized trials. Eur. Heart J..

[114] Dalkara T., Østergaard L., Heusch G., Attwell D. (2025). Pericytes in the brain and heart: Functional roles and response to ischaemia and reperfusion. Cardiovasc. Res..

[115] Kleinbongard P., Heusch G. (2022). A fresh look at coronary microembolization. Nat. Rev. Cardiol..

[116] Kloner R.A., Ganote C.E., Jennings R.B. (1974). The ‘No-Reflow’ Phenomenon after Temporary Coronary Occlusion in the Dog. J. Clin. Investig..

[117] Bière L., Donal E., Terrien G., Kervio G., Willoteaux S., Furber A., Prunier F. (2014). Longitudinal Strain Is a Marker of Microvascular Obstruction and Infarct Size in Patients with Acute ST-Segment Elevation Myocardial Infarction. PLoS ONE.

[118] O’Farrell F.M., Mastitskaya S., Hammond-Haley M., Freitas F., Wah W.R., Attwell D. (2017). Capillary pericytes mediate coronary no-reflow after myocardial ischaemia. eLife.

[119] Alarcon-Martinez L., Yilmaz-Ozcan S., Yemisci M., Schallek J., Kılıç K., Villafranca-Baughman D., Can A., Di Polo A., Dalkara T. (2019). Retinal ischemia induces α-SMA-mediated capillary pericyte contraction coincident with perivascular glycogen depletion. Acta Neuropathol. Commun..

[120] Galli M., Niccoli G., De Maria G., Brugaletta S., Montone R.A., Vergallo R., Benenati S., Magniani G., D’aMario D., Porto I. (2024). Coronary microvascular obstruction and dysfunction in patients with acute myocardial infarction. Nat. Rev. Cardiol..

[121] Methner C., Cao Z., Mishra A., Kaul S. (2021). Mechanism and potential treatment of the ‘no reflow’ phenomenon after acute myocardial infarction: Role of pericytes and GPR39. Am. J. Physiol. Heart Circ. Physiol..

[122] Li Q., Guo Z., Wu C., Tu Y., Wu Y., Xie E., Yu C., Sun W., Li X., Zheng J. (2022). Ischemia preconditioning alleviates ischemia/reperfusion injury-induced coronary no-reflow and contraction of microvascular pericytes in rats. Microvasc. Res..

[123] Reinstadler S.J., Stiermaier T., Reindl M., Feistritzer H.-J., Fuernau G., Eitel C., Desch S., Klug G., Thiele H., Metzler B. (2019). Intramyocardial haemorrhage and prognosis after ST-elevation myocardial infarction. Eur. Heart J. Cardiovasc. Imaging.

[124] Ishida T., Yarimizu K., Gute D.C., Korthuis R.J. (1997). MECHANISMS OF ISCHEMIC PRECONDITIONING. Shock.

[125] Yellon D.M., Beikoghli Kalkhoran S., Davidson S.M. (2023). The RISK pathway leading to mitochondria and cardioprotection: How everything started. Basic Res. Cardiol..

[126] Kleinbongard P. (2023). Perspective: Mitochondrial STAT3 in cardioprotection. Basic Res. Cardiol..

[127] Abd-Elfattah A.S., Ding M., Wechsler A.S. (1995). Intermittent aortic crossclamping prevents cumulative adenosine triphosphate depletion, ventricular fibrillation, and dysfunction (stunning): Is it preconditioning?. J. Thorac. Cardiovasc. Surg..

[128] Alkhulaifi A., Yellon D., Pugsley W. (1994). Preconditioning the human heart during aorto-coronary bypass surgery. Eur. J. Cardio-Thorac. Surg..

[129] Traverse J.H., Swingen C.M., Henry T.D., Fox J., Wang Y.L., Chavez I.J., Lips D.L., Lesser J.R., Pedersen W.R., Burke N.M. (2019). NHLBI-Sponsored Randomized Trial of Postconditioning During Primary Percutaneous Coronary Intervention for ST-Elevation Myocardial Infarction. Circ. Res..

[130] Thielmann M., Kottenberg E., Kleinbongard P., Wendt D., Gedik N., Pasa S., Price V., Tsagakis K., Neuhäuser M., Peters J. (2013). Cardioprotective and prognostic effects of remote ischaemic preconditioning in patients undergoing coronary artery bypass surgery: A single-centre randomised, double-blind, controlled trial. Lancet.

[131] Hausenloy D.J., Mwamure P.K., Venugopal V., Harris J., Barnard M., Grundy E., Ashley E., Vichare S., Di Salvo C., Kolvekar S. (2007). Effect of remote ischaemic preconditioning on myocardial injury in patients undergoing coronary artery bypass graft surgery: A randomised controlled trial. Lancet.

[132] Meybohm P., Bein B., Brosteanu O., Cremer J., Gruenewald M., Stoppe C., Coburn M., Schaelte G., Böning A., Niemann B. (2015). A Multicenter Trial of Remote Ischemic Preconditioning for Heart Surgery. N. Engl. J. Med..

[133] Zangrillo A., Musu M., Greco T., Di Prima A.L., Matteazzi A., Testa V., Nardelli P., Febres D., Monaco F., Calabrò M.G. (2015). Additive Effect on Survival of Anaesthetic Cardiac Protection and Remote Ischemic Preconditioning in Cardiac Surgery: A Bayesian Network Meta-Analysis of Randomized Trials. PLoS ONE.

[134] Crimi G., Pica S., Raineri C., Bramucci E., De Ferrari G.M., Klersy C., Ferlini M., Marinoni B., Repetto A., Romeo M. (2013). Remote Ischemic Post-Conditioning of the Lower Limb During Primary Percutaneous Coronary Intervention Safely Reduces Enzymatic Infarct Size in Anterior Myocardial Infarction. JACC Cardiovasc. Interv..

[135] Verouhis D., Sörensson P., Gourine A., Henareh L., Persson J., Saleh N., Settergren M., Sundqvist M., Tornvall P., Witt N. (2016). Effect of remote ischemic conditioning on infarct size in patients with anterior ST-elevation myocardial infarction. Am. Heart J..

[136] Davidson S.M., Ferdinandy P., Andreadou I., Bøtker H.E., Heusch G., Ibanez B., Ovize M., Schulz R., Yellon D.M., Hausenloy D.J. (2019). Multitarget Strategies to Reduce Myocardial Ischemia/Reperfusion Injury. J. Am. Coll. Cardiol..

[137] Eitel I., Stiermaier T., Rommel K.P., Fuernau G., Sandri M., Mangner N., Linke A., Erbs S., Lurz P., Boudriot E. (2015). Cardioprotection by combined intrahospital remote ischaemic perconditioning and postconditioning in ST-elevation myocardial infarction: The randomized LIPSIA CONDITIONING trial. Eur. Heart J..

[138] Sloth A.D., Schmidt M.R., Munk K., Kharbanda R.K., Redington A.N., Schmidt M., Pedersen L., Sørensen H.T., Bøtker H.E., CONDI Investigators (2014). Improved long-term clinical outcomes in patients with ST-elevation myocardial infarction undergoing remote ischaemic conditioning as an adjunct to primary percutaneous coronary intervention. Eur. Heart J..

[139] Morrow D.A., Antman E.M., Charlesworth A., Cairns R., Murphy S.A., de Lemos J.A., Giugliano R.P., McCabe C.H., Braunwald E. (2000). TIMI Risk Score for ST-Elevation Myocardial Infarction: A Convenient, Bedside, Clinical Score for Risk Assessment at Presentation. Circulation.

[140] Hadebe N., Cour M., Lecour S. (2018). The SAFE pathway for cardioprotection: Is this a promising target?. Basic Res. Cardiol..

[141] Hausenloy D.J., Iliodromitis E.K., Andreadou I., Papalois A., Gritsopoulos G., Anastasiou-Nana M., Kremastinos D.T., Yellon D.M. (2012). Investigating the Signal Transduction Pathways Underlying Remote Ischemic Conditioning in the Porcine Heart. Cardiovasc. Drugs Ther..

[142] Gong G.Q., Bilanges B., Allsop B., Masson G.R., Roberton V., Askwith T., Oxenford S., Madsen R.R., Conduit S.E., Bellini D. (2023). A small-molecule PI3Kα activator for cardioprotection and neuroregeneration. Nature.

[143] Majka M., Kleibert M., Wojciechowska M. (2021). Impact of the Main Cardiovascular Risk Factors on Plasma Extracellular Vesicles and Their Influence on the Heart’s Vulnerability to Ischemia-Reperfusion Injury. Cells.

[144] Xia J., Qu Y., Yin C., Xu D. (2015). Preoperative Rosuvastatin Protects Patients with Coronary Artery Disease Undergoing Noncardiac Surgery. Cardiology.

[145] Suades R., Padró T., Alonso R., Mata P., Badimon L. (2013). Lipid-lowering therapy with statins reduces microparticle shedding from endothelium, platelets and inflammatory cells. Thromb. Haemost..

[146] Nuhu F., Bhandari S. (2018). Oxidative Stress and Cardiovascular Complications in Chronic Kidney Disease, the Impact of Anaemia. Pharmaceuticals.

[147] Lu Y., Meng L., Wang X., Zhang Y., Zhang C., Zhang M. (2025). The Non-Traditional Cardiovascular Culprits in Chronic Kidney Disease: Mineral Imbalance and Uremic Toxin Accumulation. Int. J. Mol. Sci..

[148] Anzai T. (2018). Inflammatory Mechanisms of Cardiovascular Remodeling. Circ. J..

[149] Wang X., Li W., Zhang Y., Sun Q., Cao J., Tan N., Yang S., Lu L., Zhang Q., Wei P. (2022). Calycosin as a Novel PI3K Activator Reduces Inflammation and Fibrosis in Heart Failure Through AKT–IKK/STAT3 Axis. Front. Pharmacol..

[150] Tamsin Lisa K., Gilpin E., Ahnve S., Henning H., Ross J. (1985). Smoking status at the time of acute myocardial infarction and subsequent prognosis. Am. Heart J..

[151] Mobarrez F., Antoniewicz L., Bosson J.A., Kuhl J., Pisetsky D.S., Lundbäck M. (2014). The Effects of Smoking on Levels of Endothelial Progenitor Cells and Microparticles in the Blood of Healthy Volunteers. PLoS ONE.

[152] Cordazzo C., Petrini S., Neri T., Lombardi S., Carmazzi Y., Pedrinelli R., Paggiaro P., Celi A. (2014). Rapid shedding of proinflammatory microparticles by human mononuclear cells exposed to cigarette smoke is dependent on Ca^2+^ mobilization. Inflamm. Res..

[153] Toldo S., Mauro A.G., Cutter Z., Abbate A. (2018). Inflammasome, pyroptosis, and cytokines in myocardial ischemia-reperfusion injury. Am. J. Physiol. Heart Circ. Physiol..

[154] Fujita Y., Araya J., Ochiya T. (2015). Extracellular vesicles in smoking-related lung diseases. Oncotarget.

[155] Díaz I., Calderón-Sánchez E., Del Toro R., Ávila-Médina J., de Rojas-de Pedro E.S., Domínguez-Rodríguez A., Rosado J.A., Hmadcha A., Ordóñez A., Smani T. (2017). miR-125a, miR-139 and miR-324 contribute to Urocortin protection against myocardial ischemia-reperfusion injury. Sci. Rep..

[156] Godtfredsen S.J., Kragholm K.H., Leutscher P., Jørgensen S.H., Christensen M.K., Butt J.H., Gislason G., Køber L., Fosbøl E.L., Sessa M. (2022). Effectiveness and safety of P2Y12 inhibitors in patients with ST-segment elevation myocardial infarction undergoing percutaneous coronary intervention: A nationwide registry-based study. Eur. Heart J. Acute Cardiovasc. Care.

[157] Abe J., Vujic A., Prag H.A., Murphy M.P., Krieg T. (2024). Malonate given at reperfusion prevents post-myocardial infarction heart failure by decreasing ischemia/reperfusion injury. Basic Res. Cardiol..

[158] Sacks B., Onal H., Martorana R., Sehgal A., Harvey A., Wastella C., Ahmad H., Ross E., Pjetergjoka A., Prasad S. (2021). Mitochondrial targeted antioxidants, mitoquinone and SKQ1, not vitamin C, mitigate doxorubicin-induced damage in H9c2 myoblast: Pretreatment vs. co-treatment. BMC Pharmacol. Toxicol..

[159] Kong L., Xiong F., Sun N., Xu C., Chen Y., Yang J., Su X. (2020). CaMKIIδ inhibition protects against myocardial ischemia/reperfusion injury: Role of Beclin-1-dependent autophagy. Eur. J. Pharmacol..

[160] Panagiotou A., Trendelenburg M., Osthoff M. (2018). The Lectin Pathway of Complement in Myocardial Ischemia/Reperfusion Injury—Review of Its Significance and the Potential Impact of Therapeutic Interference by C1 Esterase Inhibitor. Front. Immunol..

[161] Riedemann N.C., Ward P.A. (2003). Complement in Ischemia Reperfusion Injury. Am. J. Pathol..

[162] Zou L., Zhu D., Gong M., Yu J. (2024). The influence of genetic predisposition to oxidative stress on painful diabetic peripheral neuropathy: A Mendelian randomization study. Cell. Mol. Biol..

[163] Márta K., Hasan P., Rodríguez-Prados M., Paillard M., Hajnóczky G. (2021). Pharmacological inhibition of the mitochondrial Ca^2+^ uniporter: Relevance for pathophysiology and human therapy. J. Mol. Cell. Cardiol..

[164] Miyazawa K., Fukuyama H., Komatsu E., Yamaguchi I. (1986). Effects of propranolol on myocardial damage resulting from coronary artery occlusion followed by reperfusion. Am. Heart J..

[165] Park H., Otani H., Oishi C., Fujikawa M., Yamashita K., Okazaki T., Sato D., Ueyama T., Iwasaka J., Yamamoto Y. (2011). Efficacy of intracoronary administration of a short-acting β-blocker landiolol during reperfusion in pigs. Int. J. Cardiol..

[166] Ramadan M.M., Al-Najjar R.A., Abady R.S., Obaid H.A., Mostafa Y.A., Al-Obeid M.T., Elmahal M. (2025). Mavacamten Cardiac Myosin Inhibitor: Clinical Applications and Future Perspectives. Cureus.

